# Supplementary material for: Heritability of the Symbiodinium community in vertically- and horizontally-transmitting broadcast spawning corals
Source: Sci Rep. 2017 Aug 15;7:8219. doi: 10.1038/s41598-017-08179-4 (PMC5557748; doi:10.1038/s41598-017-08179-4)
Supplement: Supplementary file 1 — Supplementary information [file 41598_2017_8179_MOESM1_ESM.doc]

**Supplementary material for “Heritability of the *Symbiodinium* community in vertically- and horizontally-transmitting broadcast spawning corals”.**

**­­­**

**Kate M. Quigley, Bette L. Willis and Line K. Bay.**

**Supplementary Methods**

**DNA extraction**

Genomic DNA was extracted from all samples using a modified SDS method with the addition of 20 µl Proteinese K to the extraction buffer ^1^. The extraction buffer contained the following (final concentrations indicted in parenthesis): 1.0 M Tris pH 9.0 (0.1M), 0.5 M EDTA (0.1M), 10% SDS (1%), 5.0 M NaCl (0.1M), Milli-Q Water (for 40 mL of extraction buffer, add 23.2 mL). Tissue samples were added to 750µL of buffer and lysed using a FastPrep-24 matrix 1mm silicia spheres (MPBio) three times at 30 s and 4.0ms-1 and incubated at 65°C for two hours. After incubation, samples were placed on ice and 187.5uL of 5 M KOAc (1 M) was added to each, vortexed and left on ice for 10 min. After vortexing, samples were centrifuged at 16,100 rcf for 20 min at room temperature, and the supernatant transferred to a new tube. 600 uL of 100% Isopropanol was added, the tube inverted to mix gently and let stand for 5 min. Samples were centrifuged as described above but for 15 min., and then decanted. A wash of 150μL of 70% Ethanol was added to each tube, mixed, and centrifuged as above for 5 min. The supernatant was removed and the pellet was left to dry for 5 min and then finally re-suspended in 50 μL of 10 mM Tris pH 9. Due to the small size of single eggs, the extraction buffer was decreased for *M. digitata* to 100 µL with the same reagents excluding the SDS. Steps involving KOAc, Isopoanol and Ethanol were removed and replaced with 30 sec at 37°C and then 2 min at 92°C ^2^. DNA extractions were sent to the University of Texas at Austin’s Genomics Sequencing and Analysis Facility (USA) for library preparation and paired-end sequencing on the Miseq (Illumina). ITS-2 primers for *Symbiodinium* amplification were as follows: ITS2alg-F (5'-TCGTCGGCAGCGTCAGATGTGTATAAGAGACAGGTGAATTGCAGAACTCCGTG) and ITS2alg-R (3'-TTCGTATATTCATTCGCCTCCGACAGAGAATATGTGTAGAGGCTCGGGTGCTCTG-5')^3^.

**Sequencing depth­­­**

To assess if *Symbiodinium* community diversity was estimated correctly with our sampling strategy, rarefaction curves and the number of unobserved OTUs were calculated using the packages ‘*iNext*’ and ‘*vegan*’ ^4,5^. From the juvenile samples of *A. tenuis* collected in 2012, 5.9% (of total n = 102) of samples had underestimated *Symbiodinium* community diversity. These juveniles were from three families (AB, BC, and CB), and only represented 4.5-14% of the juveniles from those families. From juveniles collected in 2013, 12.8% (of total n = 172) of samples were underestimated, comprising juveniles from 11 of the 20 families. These juveniles however were only underestimated by very few OTUs (average 2.9±0.9). All adults from 2012 and 2013 were sequenced to saturation. Based on the low number of “missing” OTUs and under-sequenced juveniles, the sampling and sequencing strategy was deemed robust for the diversity measures and heritability estimates presented here.

**Supplementary Results**

**Comparisons of *Symbiodinium* communities among 2012 families of *A. tenuis***

Juveniles from family AB had characteristically high proportions of both D1 and A3, which collectively made up ~75% of their *Symbiodinium* communities. Type A CCMP828 made up ~30-50% of reads in 33% (of total n =12) of AB juveniles, with concomitant decreases in proportional abundances of A3 (Figure 2). Three of the 12 AB juveniles had high abundances of two G4 types, *Zoanthus* and *Favia* *ex-situ* types, C90, and an F *ex-situ* type. All juveniles also had a diversity of background types from clades A, B, C, D, G, as well as *ex-situ* and “uncultured” *Symbiodinium* types, with, on average, 25.8± 2 OTUs per sample. Juveniles from family AC followed a similar pattern of high diversity (19.8± 2.5). Although C1 was never the dominant type in AB juveniles, 25% (of total n =8) of AC juveniles were dominated by C1. The remaining juveniles in this family were dominated by D1 and CCMP828, with A3, C1, an “uncultured” type (OTU7), and a D type (OTU6) also abundant in some juveniles. Juveniles from family AD also had many background types at very low abundance (average OTUs: 23±2), although the dominant OTUs (A3, D1 and CCMP828) were found in the same pattern as in families AB and AC. Compared to those families however, type C1 was in much lower and D1a in much higher proportional abundance in these families, respectively.

BC juveniles had, on average, 22±1.3 OTUs, with individuals dominated by combinations of D1, CCMP828, C1, A3 and background types D1a, CCMP828, D1, and C1. Again, many background OTUs were recovered (n = 106), including 24 A types (for example: A3, A2, *microadriaticum*, A13, A4.3), type B (*S. muscatinei*), 29 C (C1, C3, C90), 12 D (D1, D1a, D), three E (*S. voratum*), 12 *ex-situ* (*Zoanthus, Scyphozoan, Amphisorus*), three F (F, F4), G4, 19 “uncultured” *Symbiodinium*, and two RCC2640 types (Figure 2). The CB family was also similar to juveniles from family BC, with on average, 26.7±1.5 OTUs per juvenile, and 216 OTUs in total retrieved from this family. Like BC, the CB family had background diversity that included types from clade A (n = 72) and C (n = 110), D, E and “uncultured” symbiont types, including: A3, *microadriaticum,* A4.3, A2, A13, C1, C90, C15 and C3. Family CB, however, had a greater diversity of different G, F and B types. Families CD and CA were also very diverse, with on average 24.7±3.5 and 27.5±7.5 OTUs per juvenile respectively.

Juveniles from family BA were most similar in *Symbiodinium* diversity (17.8±1.4) and composition to AC juveniles, with C1 dominant (~75% relative abundance) in 22% (of total n = 9) of juveniles, with the remaining individuals harbouring mixed communities of D1, CCMP828, and A3 and lower abundances of D1a (Figure 2). D type (OTU6) was again recovered in low abundances. BD family juveniles (average OTUs: 17.8±2) were also similar to these other families, with D1 and A3 dominating *Symbiodinium* communities in conjunction with CCMP828 and C1.

**Comparisons of *Symbiodinium* communities among 2013 families of *A. tenuis***

Individual juveniles from F1 were dominated by varying abundances of multiple types belonging to: A3, C1 and D1, with some individuals also having large populations of G4, D (OTU10), *S. minitum*, and A type CCMP828 (OTU7). D1a was also found in abundances ranging from ~3-15% in many juveniles (Figure 2), with types C3, CCMP2456, G4, and B2 also present (Supplementary Table S5). The same pattern of the dominant type fluctuating among A3, C1 and D1 was found in families F10, F12, F2, F5, and F6. Family F28 was similar in composition to the preceding families, although with the addition of A13 (OTU11). Notably, dominant *Symbiodinium* types in F4 juveniles also varied among D1, C1, A3, D1a and CCMP828 types, although C1 was much less prevalent in this family, with only 17% (of total n =12) of juveniles having greater than background abundances.

Families F13, F19, F22, F23, F24, F27, and F9 had much lower diversity as juveniles from these families were dominated by fluctuating proportions of D1, C1, A3 and D1a and in two individuals, CCMP828; with very few other OTUs in background proportions compared to those found in F1,F10 and F12. Generally, families F14 and F18 were distinct from the other families, with 44-83% of juveniles (F14: of total n = 6, F18: of total n = 9) of partially dominated by new D types (F14: OTU4, F18: OTUs 10/16/95), C1 and A13 (OTU11). These families had, on average, the highest number of OTUs per juvenile (F14: 34.7 ± 11.9, F18: 25.2 ± 5.3), and the greatest number of OTUs recovered across all juveniles (F14: 138 total OTUs, F18: 140) (Supplementary Table S5). Four juveniles (14cbr1, 14cbr2, 18b.1r1N2, 18br1) were particularly diverse, with 45 - 84 OTUs detected, and “uncultured” *Symbiodinium* types such as Zoox23-OTU9 at particularly high abundance and diversity in these families.

The remaining families (F8, F15, and F17) had juveniles that were similar to both the first and second group of families described. For example, 50% of juveniles from F8 (of total n = 6) and 57% from F15 (of total n = 7) exhibited the characteristic C1, D1, D1a, A3 mix, whilst the remaining juveniles strongly resembled families with high OTU diversity and were typified by *S.* *psygmophilum*, *S. natans, S. voratum, S. minutum*, C91, G4, HI-0609, and “uncultured” or *ex-situ* types. F17 was similar, although additional *Symbiodinium* diversity also included A2 and sediment derived A-type *Symbiodinium* (OTU361).

**Comparisons of *Symbiodinium* communities among families of *M. digitata* eggs**

The proportional abundance of C15 (OTU1) was lower than the average C15 abundance in eggs in three of dam 29 eggs (91.2-93.7% C15); concomitantly, proportional abundances increased in a large diversity of other types, with types A3 (OTU5), D1 (OTU3) and two “uncultured” *Symbiodinium* types (OTU9 and 14) more abundant than in eggs from other dams. Eggs from dam 11 had much less of the *ex-situ Amphisorus* type; instead background abundances were made up of C1, B1, two A types (A3, CCMP828), and D1, which were all more prevalent in dam 11. The only OTUs that differed significantly (BF p-adj < 0.05) in abundance between egg families were C1_5-OTU10, “uncultured”-OTU9 and 14, *Amphisorus*-OTU2, and C1_4-OTU8 (Figure 3, Supplementary Table S6).

Eggs from dams 24, 7, 8, and 9 were similar in their background diversity, associating with an *ex-situ Amphisorus* type (OTU2), D1 (OTU3), D1a (OTU6), C1 (OTU8), A3 (OTU5) and another C15 type (OTU50) (Figure 4, Supplementary Table S6). D1a was found in a total of 15 eggs, but only one dam (dam nine) with a majority of these eggs (four eggs). Eggs from dams 11, 26, 28 and 32 had the OTUs outlined above, as well as different A (CCMP828-OTU12, but not eggs from dam 32) and C1 types (OTU10), and their own unique types per egg family (Figure 4, Supplementary Table S1). Unique types detected in eggs included B1-OTU31 (in eggs from dam 11), “uncultured” type-OTU59 (dam 26), *S. microadriaticum*-OTU29 (dam 28), and C/D/*ex-situ* types (dam 32). Whilst OTU diversity was generally low in eggs, eggs from dam 29 had very high diversity (although dam 29 diversity was low), with four of 12 eggs having 31-65 OTUs, two having 11-13 OTUs, and the remaining six eggs having 2-5 OTUs (Figure 4, Supplementary Table S6). Furthermore, particular eggs from this dam were distinct from the other symbiont communities in eggs (Figure 3, Figure 4, Supplementary Table S7), most likely due to the large number (n = 61) of “uncultured” types.

**Table S1.** Gamete cross design for *Acropora tenuis* juveniles from spawning season 2012. Colonies A and B were collected from Orpheus Island (OI), and colonies C and D were collected from Princess Charlotte Bay (PCB). Numbers in parenthesis following each cross are the number of individual juveniles sequenced per cross. Parent colonies collected and crossed from Orpheus are annotated OI, and colonies from Princess Charlotte Bay are PCB.

|  | **Sire** | | | | |
| --- | --- | --- | --- | --- | --- |
| **Dam** |  | **A** | **B** | **C** | **D** |
|  | **A** | - | BA (9) | CA (2) | - |
|  | **B** | AB (12) | - | CB (29) | - |
|  | **C** | AC (8) | BC (22) | - | - |
|  | **D** | AD (12) | BD (5) | CD (3) | - |

**Table S2.** Summary of the 25 gamete crosses performed, of which individuals from 20 families survived the larval stage, settled as juveniles and survived in the field. Numbers in parenthesis represent the number of juveniles sequenced per family. Parent colonies collected and crossed from Orpheus are annotated O, and colonies from Princess Charlotte Bay are W.

|  | **Sire** | | | | | | | | |
| --- | --- | --- | --- | --- | --- | --- | --- | --- | --- |
|  |  | **O4** | **O6** | **O3** | **O5** | **W11** | **W10** | **W7** | **W5** |
| **Dam** | **O4** |  | F1  (13) |  |  | F2  (12) |  |  | F4 (12) |
|  | **O6** |  |  | F5 (21) |  |  | F6  (14) |  |  |
|  | **O3** | F8  (6) |  |  |  | F9  (8) | F10 (13) |  |  |
|  | **O5** | F12 (12) | F13 (11) | F14 (6) |  | F15  (7) |  |  |  |
|  | **W11** |  | F17  (5) |  |  |  | F18  (9) |  | F19 (6) |
|  | **W10** | F26 (NA) |  |  | F21 (NA) | F22  (6) |  | F23 (1) | F24 (3) |
|  | **W7** |  |  |  | F25 (NA) | F29 (NA) |  |  |  |
|  | **W5** |  | F27  (5) | F28 (2) |  |  |  | F30 (NA) |  |

**Table S3. Summary of the sequencing performed for *A. tenuis* and *M. digitata* species. Samples were either juveniles (J), eggs (E), or adults (A). *Gamete crosses involved eight parental colonies (four from Wilkie and four from Orpheus); three replicates per colony were sequenced for Orpheus parents (n = 12 samples total), and one per Wilkie colony (n = four samples total), totalling 16 adult samples sequenced.**

| **Species** | **Year** | **Samples** | **Families**  **(N°)** | **Average reads/sample ± SE** | **Total cleaned reads** | **Average cleaned reads/sample ± SE** | **OTU N°** |
| --- | --- | --- | --- | --- | --- | --- | --- |
| *A. tenuis* | 2012 | 106  (102 J, 4 A) | 9 | 83,595±  2425 | 6,027,635 | 56,864±  2009 | 422 |
| *A. tenuis* | 2013 | 188  (172 J, 16 A*) | 25 | 72,107±  4329 | 6,873,216 | 36,560±  2922 | 568 |
| *M. digitata* | 2015 | 108  (99 E, 9 A) | 9 | 15,365±  817 | 1,398,184 | 12,946±  710 | 101 |

**Table S4.** Summary of sequencing results for *A. tenuis* from 2012 and 2013 crosses (post clean-up and E-value filters).

| **Year** | **2012** |  |  | **2013** |  |  |
| --- | --- | --- | --- | --- | --- | --- |
|  | **Sum reads** | **Percent (%)** | **OTUs** | **Sum reads** | **Percent (%)** | **OTUs** |
| **A** | 2,515,240 | 41.73 | 72 | 1,914,858 | 28.1 | 88 |
| **B** | 2,845 | 0.05 | 17 | 51,591 | 0.75 | 18 |
| **C** | 839,606 | 13.93 | 110 | 2,154,506 | 31.35 | 119 |
| **D** | 2,566,176 | 42.57 | 44 | 2,454,930 | 35.72 | 54 |
| **E** | 1,197 | 0.02 | 8 | 593 | 0.01 | 12 |
| **F** | 8,370 | 0.14 | 27 | 9,587 | 0.14 | 39 |
| **G** | 20,768 | 0.34 | 8 | 73,212 | 1.07 | 16 |
| ***Ex-situ*** | 15,036 | 0.25 | 54 | 40,178 | 0.58 | 62 |
| **“uncultured”** | 58,388 | 0.97 | 78 | 156,452 | 2.28 | 157 |
| **RCC2640** | 9 | 0.00 | 4 | 0 | 0.00 | 0 |
| **H** | 0 | 0.00 | 0 | 17,201 | 0.0001 | 1 |
| **I** | 0 | 0.00 | 0 | 108 | 0.00 | 2 |
| **TOTAL** | 6,027,635 | 100.00 | 422 | 6,873,216 | 100.00 | 568 |

**Table S5.** OTU diversity retrieved per family of *Acropora tenuis* juveniles in 2013. Bolded values below family name represent the total number of OTUs retrieved from that family.

| **Family (F-)/Adult (O- or W-)** | **Clade A** | **Clade C** | **Clade D** | **Clades B/*Ex-situ*/G/H/”uncultured”** |
| --- | --- | --- | --- | --- |
| F1  (**73**) | A3_OTU3, A3_OTU408, A3_OTU665, A3_OTU725, A3_OTU736, A3_OTU799, A3_OTU491, CCMP828_OTU7, CCMP2456_OTU73, CCMP2456_OTU457, CCMP828_OTU458, CCMP2456_OTU558,  12979_OTU638, 1631_OTU119, 65_OTU369, | C_OTU137, C_OTU232, C1_OTU1, C1_OTU113, C1_OTU237, C1_OTU326, C1_OTU370, C1_OTU597, C1_OTU549, C1_OTU559, C1_OTU567 C1_OTU661  C1_OTU743  C1_OTU776, C1_OTU121  C1_OTU803, Two0504-8_OTU269, C3_OTU637, C3_OTU347, SC13.7_OTU36 | D1_OTU2, D_OTU4, D1a_OTU6, D_OTU10, D1a_OTU142, D_OTU373, D1_OTU593 D1a_OTU727 D1a_OTU756, | minutum_OTU45, psygmophilum_B2_1635_OTU13, psygmophilum_B2_1636_OTU124, Zoanthus_OTU21, Zoanthus_OTU18, Scyphozoan_medusae1Sy23_OTU22, scyphozoan_medusae_1Sy24_OTU120, zoox23_OTU23, zoox23_OTU19, zoox23_OTU156, zoox23_OTU377, zoox23_OTU253, “uncultured”_OTU177, “uncultured”_OTU30, “uncultured”_OTU181, “uncultured”_OTU382, “uncultured”_OTU12, “uncultured”_OTU409, “uncultured”_OTU514, “uncultured”_OTU578, “uncultured”_OTU590, “uncultured”_OTU639, “uncultured”_OTU104, “uncultured”_OTU672, “uncultured”_OTU103, G4_OTU68,  G4_OTU730, G6_OTU8, F3.2_OTU96 kawagutii_OTU14 |
| F2  (**95**) | A3_OTU3, CCMP828_OTU7, CCMP2456_OTU73, A4_OTU165, A3_OTU238, A3_OTU362, A3_OTU404, A3_OTU408, CCMP828_OTU458, CCMP2456_OTU558, A13_OTU594, A3_OTU665, A3_OTU674, A3_OTU725, A3_OTU799 | C_OTU232, C_OTU63  C1_OTU1, C1_OTU121 C1_OTU145, C1_OTU236,C1_OTU237, C1_OTU242, C1_OTU287, C1_OTU432,  C1_OTU440,  C1_OTU448,  C1_OTU492,  C3_OTU347, C1_OTU641, C1_OTU661, C1_OTU724, C1_OTU669, C1_OTU755, C1_OTU567, C1_OTU762, SC13.7_OTU76, SC13.7_OTU422 | D_OTU37, D1_OTU2 D_OTU4,D_OTU5,D1D1a_OTU6, D_OTU10, , D_OTU16, D1a_OTU756, D1a_OTU142, D_OTU331, D1a_OTU748, D1a_OTU737 | psygmophilum_B2_1635_OTU13, psygmophilum_B2_1636_OTU124, minutum_OTU45, muscatinei_OTU17, zoox23_OTU253,  zoox23_OTU335,  zoox23_OTU371,  zoox23_OTU403, G2b_OTU94, G4_OTU68,  zoox23_OTU443, zoox23_OTU59, zoox23_OTU85, zoox23_OTU46, kawagutii_OTU14, zoox23_OTU561, zoox23_OTU19, zoox23_OTU591,zoox23_OTU592, zoox23_OTU615 zoox23_OTU750, zoox23_OTU23, zoox23_OTU643,  Zoanthus_OTU141, Zoanthus_OTU352, Zoanthus_OTU49 Zoanthus_OTU26, scyphozoan_medusae_1Sy24_OTU595,Zoanthus_OTU18, scyphozoan_medusae_1Sy24_ OTU84, scyphozoan_medusae_1Sy24_OTU39,  “uncultured”_OTU164, “uncultured”_OTU130, “uncultured”_OTU198,  “uncultured”_OTU327, “uncultured”_OTU382, “uncultured”_OTU430, “uncultured”_OTU514, “uncultured”_OTU461, “uncultured”_OTU104, “uncultured”_OTU97,  “uncultured”_OTU33, “uncultured”_OTU577,  HI-0609_OTU24 |
| F4  (**85**) | A3_OTU3, CCMP828_OTU7, Oku16_sand_OTU53, CCMP2456_OTU73, A3_OTU408, A3_OTU238, CCMP828_OTU458, CCMP2456_OTU558, A_OTU574, CCMP2456_OTU587, A3_OTU665, A3_OTU725, CCMP828_OTU746, A3_OTU799 | C1_OTU1, SC13.7_OTU76, C1_OTU79, C1_OTU86 C1_OTU113, C1_OTU121, C_OTU137, C1_OTU236, C1_OTU281, C3_OTU302, C1_OTU326, C1_OTU429, C1_OTU432, C1_OTU237, C1_OTU492, C33_OTU508, C1_OTU547, C1_OTU557, C1_OTU633, C1_OTU649, SC13.7_OTU670, PtMiI1-9_OTU642, OTcH-2_OTU659 | D1_OTU2, D_OTU4, D1a_OTU6, D_OTU10, , D_OTU37, D_OTU101, D_OTU126, D1a_OTU142, D_OTU373, D_OTU374, D1_OTU593, D1_OTU647, D_OTU678, D1a_OTU737, D1a_OTU727, D_OTU16 | G6_OTU8, “uncultured”_OTU12, psygmophilum_B2_1635_OTU13, Zoanthus_OTU18, “uncultured”_OTU27, Zoanthus_OTU28, “uncultured”_OTU30, “uncultured”_OTU33, minutum_OTU45, “uncultured”_OTU55, scyphozoan_medusae_1Sy24_OTU84, “uncultured”_OTU103, “uncultured”_OTU104, zoox23_OTU156, psygmophilum_B2_1637_OTU157, “uncultured”_OTU177, “uncultured”_OTU181, zoox23_OTU195, “uncultured”_OTU214, zoox23_OTU227, Zoanthus_OTU352, “uncultured”_OTU382, voratum_OTU406, “uncultured”_OTU409, HI-0509_OTU427, “uncultured”_OTU430, “uncultured”_OTU455, zoox23_OTU495, 13478_OTU530, Amphisorus_OTU576, scyphozoan_medusae_1Sy24_OTU596 kawagutii_OTU14 |
| F5  (**116**) | A3_OTU3, CCMP828_OTU7, CCMP2455_OTU50, A2_JCUSG-1_OTU66, CCMP2456_OTU73, CCMP2457_OTU173, A3_OTU799, A3_OTU238, A3_OTU408, A3_OTU725,  A3_OTU665, CCMP2456_OTU558 | C1_OTU1, SC13.7_OTU36, C_OTU63, C1_OTU113, C1_OTU86, C1_OTU121, C_OTU232, C_OTU137, C1_OTU249, C1_OTU250, C_OTU140, C15_OTU340, C1_OTU79, C1_OTU237, C3_OTU347, C1_OTU432, C1/C3_Two0501-8_OTU439, C1_OTU440, C1_OTU762, C1_OTU803, C3_OTU734, C1_OTU738, C1_OTU661, C33_OTU508, C1_OTU567, CCMP2456_OTU457, CCMP828_OTU458 | D1_OTU2, D_OTU5, D1a_OTU6, D_OTU10, , D_OTU16, D_OTU58, D_OTU101, D1a_OTU142, D_OTU187, D_OTU149, D_OTU331 D_OTU373, D_OTU374, D_OTU426, D1a_OTU756, D1a_OTU727, D_OTU678 | kawagutii_OTU14, G6_OTU8, “uncultured”_OTU12, psygmophilum_B2_1635_OTU13, Zoanthus_OTU18, zoox23_OTU19, Zoanthus_OTU21, Scyphozoan_medusae1Sy23_OTU22, zoox23_OTU23, zoox23_OTU25, Zoanthus_OTU26, “uncultured”_OTU27, Zoanthus_OTU28, “uncultured”_OTU33, scyphozoan_medusae_1Sy24_OTU34 Montipora_OTU38, “uncultured”_OTU42, minutum_OTU45, zoox23_OTU46, Zoanthus_OTU49, “uncultured”_OTU55, 1679_OTU61, voratum_OTU64, G4_OTU68, scyphozoan_medusae_1Sy24_OTU84, F3.2_OTU96, “uncultured”_OTU103, “uncultured”_OTU104, scyphozoan_medusae_1Sy24_OTU120, psygmophilum_B2_1636_OTU124, “uncultured”_OTU130, zoox23_OTU143, “uncultured”_OTU164, “uncultured”_OTU177, “uncultured”_OTU181, voratum_OTU216, 1679_OTU285, nr-i4_OTU301, “uncultured”_OTU306, Zoanthus_OTU316, Scyphozoan_medusae1Sy23_OTU333, zoox23_OTU342, 04-218-SCI.01_OTU368, “uncultured”_OTU382, “uncultured”_OTU402, zoox23_OTU403, “uncultured”_OTU430, psygmophilum_OTU453, Zoanthus_OTU454, scyphozoan_medusae_1Sy24_OTU489, zanpa_OTU498, “uncultured”_OTU514, Zoanthus_OTU545, 13478_OTU548, OTcH-2_OTU659, , “uncultured”_OTU663, Amphisorus_OTU666, psygmophilum_OTU671, scyphozoan_medusae_1Sy24_OTU729, “uncultured”_OTU754 |
| F10  (**112**) | A2_JCUSG-1_OTU66  A2_OTU215  A3_OTU128  A3_OTU291  A3_OTU3  A3_OTU309  A3_OTU408  A3_OTU476  A3_OTU491  A3_OTU665  A3_OTU725  A3_OTU799 CCMP2455_OTU50  CCMP2456_OTU163  CCMP2456_OTU558  CCMP2456_OTU73  CCMP2456_OTU757  CCMP828_OTU458  CCMP828_OTU664  CCMP828_OTU7 | C_OTU137  C1/C3_Two0501-8_OTU439  C1_OTU1  C1_OTU113  C1_OTU121  C1_OTU204  C1_OTU241  C1_OTU381  C1_OTU383  C1_OTU412  C1_OTU419  C1_OTU440  C1_OTU567  C1_OTU589  C1_OTU661  C1_OTU762  C1_OTU79  C1_OTU803  C1v1d_OTU355 SC13.7_OTU753  SC13.7_OTU76 | D_OTU10  D_OTU16  D_OTU328  D_OTU373  D_OTU4  D_OTU5  D1.2_OTU361  D1_OTU2  D1a_OTU142  D1a_OTU6  D1a_OTU737 | 1363_OTU353  1679_OTU174  1679_OTU61  1681_OTU102  1681_OTU303  Amphisorus_OTU134 F4.5_OTU93  G4_OTU68  HI-0509_OTU109  HI-0509_OTU139  HI-0509_OTU334  HI-0509_OTU427  HI-0609_OTU24, minutum_OTU385  minutum_OTU45 psygmophilum_B2_1635_OTU13  psygmophilum_B2_1636_OTU124  psygmophilum_B2_1637_OTU157  psygmophilum_OTU330 scyphozoan_medusae_1Sy24_OTU44  scyphozoan_medusae_1Sy24_OTU501  scyphozoan_medusae_1Sy24_OTU84  Scyphozoan_medusae1Sy23_OTU22  “uncultured”_OTU104  “uncultured”_OTU12  “uncultured”_OTU164  “uncultured”_OTU177  “uncultured”_OTU181  “uncultured”_OTU198  “uncultured”_OTU30  “uncultured”_OTU33  “uncultured”_OTU42  “uncultured”_OTU514  “uncultured”_OTU55  “uncultured”_OTU639  “uncultured”_OTU751  “uncultured”_OTU83  “uncultured”_OTU97  Zoanthus_OTU144  Zoanthus_OTU21  Zoanthus_OTU224  Zoanthus_OTU26  Zoanthus_OTU28  Zoanthus_OTU352  Zoanthus_OTU49  zoox23_OTU155  zoox23_OTU170  zoox23_OTU186  zoox23_OTU19  zoox23_OTU195  zoox23_OTU23  zoox23_OTU272  zoox23_OTU346  zoox23_OTU438  zoox23_OTU443  zoox23_OTU59  zoox23_OTU614  zoox23_OTU731  zoox23_OTU9 kawagutii_OTU14 |
| F6  (**107**) | A_OTU574  A_OTU69  A2_JCUSG-1_OTU66  A3_OTU110  A3_OTU3  A3_OTU408  A3_OTU665  A3_OTU725  A3_OTU799 CCMP2456_OTU457  CCMP2456_OTU558  CCMP2456_OTU73  CCMP828_OTU458  CCMP828_OTU656  CCMP828_OTU664  CCMP828_OTU7  CCMP828_OTU746 | C_OTU232  C1_OTU1"  C1_OTU113  C1_OTU121  C1_OTU145  C1_OTU287  C1_OTU336  C1_OTU363  C1_OTU381  C1_OTU423  C1_OTU440  C1_OTU448  C1_OTU459  C1_OTU463  C1_OTU467  C1_OTU496  C1_OTU499  C1_OTU624  C1_OTU633  C1_OTU79  C91_OTU484  SC13.7_OTU76 | D_OTU16  D_OTU179  D_OTU328  D_OTU372  D_OTU4  D1_OTU2  D1a_OTU142  D1a_OTU6 | 1631_OTU119  kawagutii_OTU141681_OTU102 G2_OTU265  G4_OTU68  G6_OTU47  G6_OTU8  HI-0609_OTU24  HI-0609_OTU465 minutum_OTU45  Montipora_OTU38  natans_OTU99  nr-i2_OTU235  psygmophilum_B2_1635_OTU13  psygmophilum_B2_1636_OTU124  scyphozoan_medusae_1Sy24_OTU34  scyphozoan_medusae_1Sy24_OTU660  scyphozoan_medusae_1Sy24_OTU84  Scyphozoan_medusae1Sy23_OTU333  Scyphozoan_medusae1Sy23_OTU653  “uncultured”_OTU103  “uncultured”_OTU104  “uncultured”_OTU12  “uncultured”_OTU130  “uncultured”_OTU164  “uncultured”_OTU177  “uncultured”_OTU239  “uncultured”_OTU27  “uncultured”_OTU278  “uncultured”_OTU33  “uncultured”_OTU42  “uncultured”_OTU430  “uncultured”_OTU477  “uncultured”_OTU514  “uncultured”_OTU55  “uncultured”_OTU588  “uncultured”_OTU598  “uncultured”_OTU608  “uncultured”_OTU640  voratum_OTU70  Zoanthus_OTU178  Zoanthus_OTU21  Zoanthus_OTU224  Zoanthus_OTU234  Zoanthus_OTU26  Zoanthus_OTU28  Zoanthus_OTU298  Zoanthus_OTU32  Zoanthus_OTU352  Zoanthus_OTU49  zoox23_OTU155  zoox23_OTU19  zoox23_OTU227  zoox23_OTU228  zoox23_OTU23  zoox23_OTU25  zoox23_OTU392  zoox23_OTU59  zoox23_OTU592  zoox23_OTU85 |
| F8  (**86**) | A2_JCUSG-1_OTU66  A3_OTU238  A3_OTU3  A3_OTU309  A3_OTU354  A3_OTU476  A3_OTU736  A4.3_OTU466 CCMP2455_OTU50  CCMP2456_OTU163  CCMP828_OTU254  CCMP828_OTU7 minutum_OTU45 | C_OTU534  C1_OTU1  C1_OTU113  C1_OTU121  C1_OTU167  C1_OTU273  C1_OTU544  C1_OTU550  C1_OTU740  C1v1d_OTU355  C3_OTU637  C91_OTU484 | D_OTU5  D_OTU575  D_OTU655  D1.2_OTU361  D1_OTU2  D1a_OTU6 | 13467_OTU106  13478_OTU662  1363_OTU353  1679_OTU174  1679_OTU247  1679_OTU490  1679_OTU98 Amphisorus_OTU304  Amphisorus_OTU487 kawagutii_OTU14  F4.5_OTU93  G4_OTU68  G6_OTU47  G6_OTU8  HI-0509_OTU109  HI-0509_OTU118  HI-0509_OTU139  HI-0509_OTU334  HI-0609_OTU182  HI-0609_OTU24 muscatinei_OTU133  natans_OTU99  psygmophilum_B2_1635_OTU13  psygmophilum_B2_1636_OTU124  psygmophilum_B2_1637_OTU157  psygmophilum_B2_1638_OTU286  psygmophilum_B2_1639_OTU747  scyphozoan_medusae_1Sy24_OTU84  “uncultured”_OTU103  “uncultured”_OTU104  “uncultured”_OTU180  “uncultured”_OTU197  “uncultured”_OTU260  “uncultured”_OTU30  “uncultured”_OTU31  “uncultured”_OTU33  “uncultured”_OTU672  “uncultured”_OTU742  “uncultured”_OTU83  “uncultured”_OTU97  voratum_OTU216  voratum_OTU401  voratum_OTU431  voratum_OTU70  Zoanthus_OTU158  Zoanthus_OTU178  Zoanthus_OTU224  Zoanthus_OTU28  Zoanthus_OTU32  Zoanthus_OTU49  zoox23_OTU170  zoox23_OTU186  zoox23_OTU19  zoox23_OTU195  zoox23_OTU731 |
| F9  (**52**) | A2_OTU215  A3_OTU3  A3_OTU674  A3_OTU725  A4.3_OTU114  Amami1f_OTU787  CCMP2455_OTU50  CCMP828_OTU458  CCMP828_OTU7  CCMP828_OTU746  microadriaticum_OTU580  microadriaticum_OTU683  microadriaticum_OTU760  microadriaticum_OTU788 | C_OTU140  C_OTU232  C_OTU63  C1_OTU1  C1_OTU121  C1_OTU167  C1_OTU249  C1_OTU661 | D_OTU10  D_OTU101  D_OTU179  D_OTU331  D_OTU37  D_OTU4  D1_OTU2  D1a_OTU6  D1a_OTU756 | 1681_OTU303  Amphisorus_OTU134  B16_Z1_OTU296  G4_OTU68  HI-0609_OTU24  muscatinei_OTU17  scyphozoan_medusae_1Sy24_OTU84  Scyphozoan_medusae1Sy23_OTU311  Scyphozoan_medusae1Sy23_OTU653  “uncultured”_OTU104  “uncultured”_OTU181  “uncultured”_OTU33  voratum_OTU692  voratum_OTU705  Zoanthus_OTU117  Zoanthus_OTU178  Zoanthus_OTU32  Zoanthus_OTU49  Zoanthus_OTU809  zoox23_OTU195  zoox23_OTU493 |
| F10  (**112**) | A2_JCUSG-1_OTU66  A2_OTU215  A3_OTU128  A3_OTU291  A3_OTU3  A3_OTU309  A3_OTU408  A3_OTU476  A3_OTU491  A3_OTU665  A3_OTU725  A3_OTU799 CCMP2455_OTU50  CCMP2456_OTU163  CCMP2456_OTU558  CCMP2456_OTU73  CCMP2456_OTU757  CCMP828_OTU458  CCMP828_OTU664  CCMP828_OTU7  HI-0509_OTU109  HI-0509_OTU139  HI-0509_OTU334  HI-0509_OTU427  HI-0609_OTU24 | C_OTU137  C1/C3_Two0501-8_OTU439  C1_OTU1"  C1_OTU113  C1_OTU121  C1_OTU204  C1_OTU241  C1_OTU381  C1_OTU383  C1_OTU412  C1_OTU419  C1_OTU440  C1_OTU567  C1_OTU589  C1_OTU661  C1_OTU762  C1_OTU79  C1_OTU803  C1v1d_OTU355  SC13.7_OTU753  SC13.7_OTU76 | D_OTU10  D_OTU16  D_OTU328  D_OTU373  D_OTU4  D_OTU5  D1.2_OTU361  D1_OTU2  D1a_OTU142  D1a_OTU6  D1a_OTU737 | 1363_OTU353  1679_OTU174  1679_OTU61  1681_OTU102  1681_OTU303  Amphisorus_OTU134  F4.5_OTU93  G4_OTU68  kawagutii_OTU14  minutum_OTU385  minutum_OTU45  psygmophilum_B2_1635_OTU13  psygmophilum_B2_1636_OTU124  psygmophilum_B2_1637_OTU157  psygmophilum_OTU330  scyphozoan_medusae_1Sy24_OTU44  scyphozoan_medusae_1Sy24_OTU501  scyphozoan_medusae_1Sy24_OTU84  Scyphozoan_medusae1Sy23_OTU22  “uncultured”_OTU104  “uncultured”_OTU12  “uncultured”_OTU164  “uncultured”_OTU177  “uncultured”_OTU181  “uncultured”_OTU198  “uncultured”_OTU30  “uncultured”_OTU33  “uncultured”_OTU42  “uncultured”_OTU514  “uncultured”_OTU55  “uncultured”_OTU639  “uncultured”_OTU751  “uncultured”_OTU83  “uncultured”_OTU97  Zoanthus_OTU144  Zoanthus_OTU21  Zoanthus_OTU224  Zoanthus_OTU26  Zoanthus_OTU28  Zoanthus_OTU352  Zoanthus_OTU49  zoox23_OTU155  zoox23_OTU170  zoox23_OTU186  zoox23_OTU19  zoox23_OTU195  zoox23_OTU23  zoox23_OTU272  zoox23_OTU346  zoox23_OTU438  zoox23_OTU443  zoox23_OTU59  zoox23_OTU614  zoox23_OTU731  zoox23_OTU9 |
| F12  (**60**) | A2_JCUSG-1_OTU66  A3_OTU183  A3_OTU3  A3_OTU408  A3_OTU476  A3_OTU665  A3_OTU725  A3_OTU736  A3_OTU799  A4.3_OTU114 CCMP2456_OTU558  CCMP2456_OTU73  CCMP828_OTU458  CCMP828_OTU7  CCMP828_OTU746 | C_OTU140  C_OTU232  C_OTU63  C1/C3_Two0501-8_OTU439  C1_OTU1"  C1_OTU113  C1_OTU121  C1_OTU237  C1_OTU413  C1_OTU432  C1_OTU567  C1_OTU661  C1_OTU762  C1_OTU803  C3_OTU637 | D_OTU10  D_OTU16  D_OTU374  D_OTU4  D_OTU426  D_OTU456  D_OTU5  D_OTU678  D1_OTU2  D1a_OTU6  D1a_OTU756 | 1679_OTU61  HI-0609_OTU24  scyphozoan_medusae_1Sy24_OTU39  scyphozoan_medusae_1Sy24_OTU390  “uncultured”_OTU103  “uncultured”_OTU104  “uncultured”_OTU177  “uncultured”_OTU206  “uncultured”_OTU33  “uncultured”_OTU430  “uncultured”_OTU514  “uncultured”_OTU672  Zoanthus_OTU26  Zoanthus_OTU28  Zoanthus_OTU352  zoox21_OTU35  zoox23_OTU186  zoox23_OTU23  zoox23_OTU9 |
| F13  (**57**) | A13_OTU57  A3_OTU3  A3_OTU408  A3_OTU665  A3_OTU725  A3_OTU779  A3_OTU799  CCMP2456_OTU558  CCMP2456_OTU73  CCMP828_OTU458  CCMP828_OTU7 | 04-218-SCI.01_OTU368  C_OTU140  C_OTU232  C_OTU63  C1_OTU1"  C1_OTU121  C1_OTU237  C1_OTU567  C1_OTU633  C1_OTU661  C1_OTU688  C1_OTU762  C1_OTU767  C1_OTU79  C1_OTU803 | D_OTU10  D_OTU16  D_OTU5  D1_OTU2  D1a_OTU6  D1a_OTU727  D1a_OTU756  D1a_OTU811 | 12979_OTU625  13478_OTU184  1679_OTU174  Amphisorus_OTU67 G6_OTU8  minutum_OTU45  Palythoa_OTU568  Protopalythoa_OTU395  scyphozoan_medusae_1Sy24_OTU120  “uncultured”_OTU104  “uncultured”_OTU108  “uncultured”_OTU129  “uncultured”_OTU514  “uncultured”_OTU812  Zoanthus_OTU208  Zoanthus_OTU28  Zoanthus_OTU316  Zoanthus_OTU49  zoox23_OTU19  zoox23_OTU322  zoox23_OTU391  zoox23_OTU552  zoox23_OTU59 |
| F14  (**138**) | A13_OTU11  A2_JCUSG-1_OTU66  A3_OTU111  A3_OTU128  A3_OTU159  A3_OTU191  A3_OTU238  A3_OTU291  A3_OTU3  A3_OTU309  A3_OTU339  A3_OTU476  A3_OTU482  A3_OTU71  A3_OTU725  A3_OTU77  A3_OTU782  A4.3_OTU114  A4.3_OTU466  CCMP828_OTU7  microadriaticum_OTU772 | C_OTU140  C_OTU232  C_OTU259  C_OTU63  C1_OTU1  C1_OTU121  C1_OTU249  C1_OTU315  C1_OTU381  C1_OTU451  C1_OTU528  C1_OTU610  C1_OTU613  C1_OTU617  C1_OTU762  C1_OTU80  C1v1e_OTU716  C3_OTU529  C3_OTU690 | D_OTU10  D_OTU126  D_OTU16  D_OTU313  D_OTU4  D_OTU405  D_OTU5  D_OTU58  D_OTU678  D_OTU722  D_OTU781  D1.2_OTU207  D1.2_OTU277  D1.2_OTU312  D1_OTU2  D1a_OTU6 | 13467_OTU106  13478_OTU621  1363_OTU687  1363_OTU81  1679_OTU223  1679_OTU271  1679_OTU61  1679_OTU804  1679_OTU98  Amphisorus_OTU203  Amphisorus_OTU418  B16_OTU175  B16_OTU329  B16_Z1_OTU296  F3.2_OTU96  G2_OTU244  G2_OTU424  G2b_OTU169  G2b_OTU192  G2b_OTU94  G3.4_OTU48  G4_OTU68  G6_OTU8  HI-0509_OTU118  HI-0509_OTU138  HI-0509_OTU139  HI-0509_OTU152  HI-0509_OTU773  HI-0609_OTU210  HI-0609_OTU225  HI-0609_OTU24 mizugama_OTU91  muscatinei_OTU133  muscatinei_OTU17  natans_OTU99  Odo06_A7_OTU384  Oku03_sand_OTU205  psygmophilum_B2_1635_OTU13  scyphozoan_medusae_1Sy24_OTU34  scyphozoan_medusae_1Sy24_OTU41  “uncultured”_OTU104  “uncultured”_OTU115  “uncultured”_OTU180  “uncultured”_OTU190  “uncultured”_OTU20  “uncultured”_OTU246  “uncultured”_OTU260  “uncultured”_OTU290  “uncultured”_OTU31  “uncultured”_OTU33  “uncultured”_OTU375  “uncultured”_OTU40  “uncultured”_OTU416  “uncultured”_OTU43  “uncultured”_OTU468  “uncultured”_OTU502  “uncultured”_OTU510  “uncultured”_OTU60  “uncultured”_OTU681  “uncultured”_OTU715  “uncultured”_OTU791  “uncultured”_OTU813  “uncultured”_OTU83  “uncultured”_OTU97  voratum_OTU212  voratum_OTU263  voratum_OTU264  voratum_OTU616  Zoanthus_OTU117  Zoanthus_OTU188  Zoanthus_OTU26  Zoanthus_OTU28  Zoanthus_OTU295  Zoanthus_OTU320  Zoanthus_OTU352  Zoanthus_OTU49  zoox23_OTU156  zoox23_OTU186  zoox23_OTU276  zoox23_OTU54  zoox23_OTU731  zoox23_OTU9 |
| F15  (**131**) | A1_Mf_OTU321  A13_OTU11  A13_OTU57  A13_OTU65  A2_JCUSG-1_OTU66  A3_OTU159  A3_OTU176  A3_OTU3  A3_OTU408  A3_OTU476  A3_OTU482  A3_OTU725  A3_OTU77  A3_OTU818  CCMP2455_OTU50  CCMP2456_OTU558  CCMP2456_OTU584  CCMP2456_OTU73  CCMP828_OTU7 | C_OTU137  C_OTU140  C_OTU232  C_OTU63  C1_OTU1"  C1_OTU121  C1_OTU237  C1_OTU249  C1_OTU381  C1_OTU429  C1_OTU444  C1_OTU469  C1_OTU567  C1_OTU634  C1_OTU661  C1_OTU762  C1_OTU766  C3_OTU529  SC13.7_OTU76 | D_OTU16  D_OTU4  D_OTU5  D_OTU58  D_OTU623  D_OTU629  D_OTU678  D_OTU714  D1.2_OTU207  D1_OTU2  D1_OTU89  D1a_OTU6  D1a_OTU727 | 13467_OTU106  13478_OTU283  13478_OTU398  1363_OTU100  1363_OTU81  1679_OTU174  1679_OTU332  1679_OTU433  1679_OTU61  Amphisorus_OTU134  Amphisorus_OTU203  Amphisorus_OTU51  Amphisorus_OTU67  B16_OTU175  F4.5_OTU603  F4.5_OTU93  G2_OTU424  G2b_OTU105  G2b_OTU169  G2b_OTU94  G3.4_OTU48  G4_OTU221  G4_OTU68  G6_OTU47  G6_OTU8  H1_OTU542  HI-0509_OTU109  HI-0509_OTU118  HI-0509_OTU138  HI-0509_OTU139  HI-0609_OTU148  HI-0609_OTU182  HI-0609_OTU225  HI-0609_OTU24  mizugama_OTU91  muscatinei_OTU17  psygmophilum_OTU453  scyphozoan_medusae_1Sy24_OTU120  scyphozoan_medusae_1Sy24_OTU34  scyphozoan_medusae_1Sy24_OTU41  scyphozoan_medusae_1Sy24_OTU74  scyphozoan_medusae_1Sy24_OTU807  scyphozoan_medusae_1Sy24_OTU84  Scyphozoan_medusae1Sy23_OTU311  “uncultured”_OTU104  “uncultured”_OTU12  “uncultured”_OTU123  “uncultured”_OTU164  “uncultured”_OTU181  “uncultured”_OTU255  “uncultured”_OTU274  “uncultured”_OTU33  “uncultured”_OTU42  “uncultured”_OTU585  “uncultured”_OTU60  “uncultured”_OTU627  “uncultured”_OTU645  “uncultured”_OTU681  “uncultured”_OTU774  “uncultured”_OTU83  “uncultured”_OTU90  “uncultured”_OTU97  voratum_OTU70  zanpa_OTU612  Zoanthus_OTU117  Zoanthus_OTU28  Zoanthus_OTU352  Zoanthus_OTU49  zoox23_OTU170  zoox23_OTU186  zoox23_OTU23  zoox23_OTU231  zoox23_OTU342  zoox23_OTU380  zoox23_OTU387  zoox23_OTU400  zoox23_OTU437  zoox23_OTU54  zoox23_OTU56  zoox23_OTU59 |
| F17  (**50**) | A13_OTU11  A2_JCUSG-1_OTU66  A3_OTU3  A3_OTU665  A3_OTU725  A3_OTU799  Asand_Oku17_OTU631  CCMP2456_OTU73  CCMP828_OTU7 | C_OTU162  C_OTU232  C_OTU63  C1_OTU1"  C1_OTU121  C1_OTU145  C1_OTU669 | D_OTU4  D_OTU5  D1_OTU2  D1a_OTU6 | 13478_OTU184  13478_OTU310  13478_OTU512  Amphisorus_OTU51  Amphisorus_OTU67  G6_OTU8  HI-0509_OTU139  scyphozoan_medusae_1Sy24_OTU34  scyphozoan_medusae_1Sy24_OTU41  scyphozoan_medusae_1Sy24_OTU84  Scyphozoan_medusae1Sy23_OTU311  “uncultured”_OTU104  “uncultured”_OTU108  “uncultured”_OTU185  “uncultured”_OTU198  “uncultured”_OTU33  “uncultured”_OTU399  “uncultured”_OTU42  Zoanthus_OTU26  zoox23_OTU150  zoox23_OTU156  zoox23_OTU19  zoox23_OTU23  zoox23_OTU248  zoox23_OTU262  zoox23_OTU380  zoox23_OTU403  zoox23_OTU551  zoox23_OTU59  zoox23_OTU750 |
| F18  (**140**) | A13_OTU11  A13_OTU471  A2_JCUSG-1_OTU66  A3_OTU183  A3_OTU200  A3_OTU291  A3_OTU3  A3_OTU339  A4.3_OTU114  A4_OTU165  CCMP2455_OTU50  CCMP2456_OTU558  CCMP2456_OTU73  CCMP828_OTU458  CCMP828_OTU7 | C_OTU137  C_OTU700  C1_OTU1  C1_OTU113  C1_OTU121  C1_OTU194  C1_OTU325  C1_OTU661  C1_OTU680  C1_OTU703  C1_OTU789  C1_OTU79  C1_OTU803  C1_OTU86  C3_OTU480 | D_OTU10  D_OTU16  D_OTU349  D_OTU4  D_OTU5  D_OTU531  D_OTU58  D_OTU628  D_OTU678  D_OTU763  D_OTU764  D_OTU805  D_OTU95  D1_OTU2  D1_OTU593  D1a_OTU6 | 13478_OTU132  13478_OTU548  1363_OTU81  1631_OTU119  1679_OTU98  1681_OTU102  B16_OTU268  F3.2_OTU96  G2b_OTU171  G2b_OTU650  G2b_OTU94  G3.4_OTU154  G4_OTU68  G6_OTU47  G6_OTU8  HI-0609_OTU24  mizugama_OTU91  muscatinei_OTU133  muscatinei_OTU699  natans_OTU122  scyphozoan_medusae_1Sy24_OTU220  scyphozoan_medusae_1Sy24_OTU294  scyphozoan_medusae_1Sy24_OTU39  Scyphozoan_medusae1Sy23_OTU125  Scyphozoan_medusae1Sy23_OTU333  “uncultured”_OTU103  “uncultured”_OTU104  “uncultured”_OTU12  “uncultured”_OTU129  “uncultured”_OTU146  “uncultured”_OTU197  “uncultured”_OTU199  “uncultured”_OTU201  “uncultured”_OTU213  “uncultured”_OTU31  “uncultured”_OTU33  “uncultured”_OTU382  “uncultured”_OTU40  “uncultured”_OTU42  “uncultured”_OTU43  “uncultured”_OTU468  “uncultured”_OTU494  “uncultured”_OTU55  “uncultured”_OTU573  “uncultured”_OTU60  “uncultured”_OTU605  “uncultured”_OTU813  “uncultured”_OTU825  “uncultured”_OTU97  Zoanthus_OTU117  Zoanthus_OTU144  Zoanthus_OTU188  Zoanthus_OTU211  Zoanthus_OTU26  Zoanthus_OTU28  Zoanthus_OTU316  Zoanthus_OTU32  Zoanthus_OTU365  Zoanthus_OTU88  zoox21_OTU35  zoox23_OTU136  zoox23_OTU15  zoox23_OTU151  zoox23_OTU156  zoox23_OTU186  zoox23_OTU19  zoox23_OTU196  zoox23_OTU218  zoox23_OTU227  zoox23_OTU23  zoox23_OTU256  zoox23_OTU272  zoox23_OTU284  zoox23_OTU293  zoox23_OTU300  zoox23_OTU377  zoox23_OTU443  zoox23_OTU46  zoox23_OTU52  zoox23_OTU537  zoox23_OTU54  zoox23_OTU554  zoox23_OTU56  zoox23_OTU569  zoox23_OTU59  zoox23_OTU592  zoox23_OTU72  zoox23_OTU731  zoox23_OTU75  zoox23_OTU750  zoox23_OTU78  zoox23_OTU82  zoox23_OTU9  zoox23_OTU92 |
| F19  (**35**) | A3_OTU222  A3_OTU3  CCMP2455_OTU50  CCMP2456_OTU558  CCMP2456_OTU73  CCMP828_OTU458  CCMP828_OTU7  CCMP828_OTU746 | C_OTU140  C_OTU232  C1_OTU1  C1_OTU113  C1_OTU121  C1_OTU571 | D_OTU16  D_OTU4  D_OTU426  D_OTU5  D1_OTU2  D1a_OTU6  D1a_OTU821 | 13478_OTU452  HI-0609_OTU601  minutum_OTU45  psygmophilum_OTU606  “uncultured”_OTU104  “uncultured”_OTU129  “uncultured”_OTU33  “uncultured”_OTU539  “uncultured”_OTU90  Zoanthus_OTU28  zoox23_OTU23  zoox23_OTU75  zoox23_OTU9  zoox23_OTU92 |
| F22  (**35**) | A3_OTU252  A3_OTU3  A3_OTU725  CCMP2456_OTU558  CCMP828_OTU7 | C_OTU140  C_OTU153  C_OTU232  C_OTU768  C1_OTU1"  C1_OTU121  C1_OTU661  C1_OTU762  C1_OTU797  C1_OTU803  SC13.7_OTU684  SC13.7_OTU76 | D_OTU331  D_OTU4  D_OTU5  D1_OTU2  D1a_OTU6  D1a_OTU727  D1a_OTU756 | 1679_OTU98  “uncultured”_OTU104  “uncultured”_OTU43  “uncultured”_OTU514  “uncultured”_OTU607  “uncultured”_OTU608  Zoanthus_OTU28  Zoanthus_OTU32  Zoanthus_OTU88  zoox21_OTU35  zoox23_OTU56 |
| F23  (**13**) | A3_OTU3  CCMP828_OTU7 | C1_OTU1"  C1_OTU649 | D1_OTU2  D1a_OTU6 | scyphozoan_medusae_1Sy24_OTU39  Zoanthus_OTU26  Zoanthus_OTU28  Zoanthus_OTU565  zoox21_OTU35  zoox23_OTU56  zoox23_OTU59 |
| F24  (**34**) | A3_OTU159  A3_OTU3  A3_OTU408  A3_OTU725  A3_OTU799  CCMP2455_OTU50  CCMP828_OTU458  CCMP828_OTU664  CCMP828_OTU7  CCMP828_OTU746 | C_OTU232  C1_OTU1"  C1_OTU121  C1_OTU815  C1_OTU86 | D_OTU16  D_OTU405  D_OTU652  D1_OTU2  D1a_OTU460  D1a_OTU6 | F3.2_OTU96  G4_OTU68  scyphozoan_medusae_1Sy24_OTU39  “uncultured”_OTU43  “uncultured”_OTU55  Zoanthus_OTU21  Zoanthus_OTU28  Zoanthus_OTU352  Zoanthus_OTU88  zoox21_OTU35  zoox23_OTU136  zoox23_OTU186  zoox23_OTU392 |
| F27  (**33**) | A3_OTU3  A3_OTU408  A3_OTU725  CCMP2456_OTU73  CCMP828_OTU458  CCMP828_OTU7  CCMP828_OTU746 | C_OTU137  C_OTU140  C_OTU232  C_OTU63  C1_OTU1"  C1_OTU121  C1_OTU567  C3_OTU523 | D_OTU4  D1_OTU2  D1a_OTU6 | 1341_OTU394 HI-0609_OTU24  minutum_OTU45  “uncultured”_OTU104  “uncultured”_OTU130  “uncultured”_OTU177  “uncultured”_OTU278  “uncultured”_OTU33  “uncultured”_OTU42  “uncultured”_OTU43  “uncultured”_OTU55  Zoanthus_OTU21  Zoanthus_OTU28  zoox23_OTU72  zoox23_OTU9 |
| F28  (**30**) | A_OTU69,  A13_OTU11,  A3_OTU3,  A3_OTU725,  CCMP828_OTU7 | C_OTU232,  C1_OTU,  C1_OTU121,  C1_OTU282,  C1_OTU345,  C1_OTU428,  C1_OTU544,  C1_OTU86, | D_OTU16,  D_OTU4,  D1_OTU2,  D1a_OTU6 | 12979_OTU625,  13478_OTU132,  Amphisorus_OTU418,  “uncultured”_OTU104,  “uncultured”_OTU177,  “uncultured”_OTU33,  “uncultured”_OTU468,  Zoanthus_OTU144,  Zoanthus_OTU21,  Zoanthus_OTU483,  zoox23_OTU186,  zoox23_OTU19,  zoox23_OTU52 |
| W10  (**10**) | A3_OTU3, CCMP828_OTU7 | C1_OTU1, C_OTU232, C1_ OTU121, C_OTU63 | D1_OTU2, D1a_OTU6 | Zoanthus_OTU32, “uncultured”_OTU104 |
| W11  (**9**) | A3_OTU3, CCMP828_OTU7 | C1_OTU1, C1_OTU121, C_OTU140 | D1_OTU2, D_OTU4, D1a_OTU6 | “uncultured”_OTU104 |
| W5  (**12**) | A3_OTU3, A13_OTU11 | C1_OTU1, C1_OTU121, C_OTU232, C1_OTU762, C1_OTU803 | D1_OTU2, D1a_OTU6 | zoox23_OTU9, “uncultured”_OTU42, “uncultured”_OTU104 |
| W7  (**17**) | A3_OTU3, CCMP828_OTU7, A13_OTU11 | C1_OTU1, C_OTU63, C1_OTU121, C_OTU140, C_OTU232, C1_OTU567, C1_OTU661, C1v1e_OTU716, C1_OTU797 | D1_OTU2 D1a_OTU6 | scyphozoan_medusae_1Sy24_OTU39, “uncultured”_OTU104, Amphisorus_OTU418 |
| O3  (**20**) | A3_OTU3, CCMP828_OTU7, A_OTU69 | C1_OTU1, C_OTU63, C1_OTU121, C_OTU140, C_OTU232, C1_OTU567, C1v1e_OTU716, C1_OTU762, C1_OTU803 | D1_OTU2, D_OTU4, D1a_OTU6 | zoox23_OTU9, “uncultured”_OTU55, “uncultured”_OTU104, Zoanthus_OTU144, Amphisorus_OTU418 |
| O4  (**15**) | A3_OTU3, CCMP828_OTU7, A_OTU69, A_OTU604 | C1_OTU1, C_OTU63, C1_OTU121, C_OTU140, C_OTU232, C_OTU800 | D1_OTU2, D_OTU5, D1a_OTU6 | “uncultured”_OTU104, Amphisorus_OTU418 |
| O5  (**19**) | A3_OTU3, CCMP828_OTU7, A_OTU69, A3_OTU476 | C1_OTU1, C_OTU63, C1_OTU121, C_OTU140, C_OTU232, C1_OTU567, C1_OTU661, C1v1e_OTU716, C_OTU800 | D1_OTU2 D_OTU4, D1a_OTU6 | “uncultured”_OTU104, Zoanthus_OTU295, Amphisorus_OTU418 |
| O6  (**20**) | A3_OTU3, A13_OTU11, A_OTU69, A3_OTU222, A_OTU604 | C1_OTU1, CCMP828_OTU7, C_OTU63, C1_OTU121, C_OTU140, C_OTU232, C1_OTU567, C_OTU800, C1_OTU803 | D1_OTU2 D_OTU4, D1a_OTU6 | zoox23_OTU19, “uncultured”_OTU104, Amphisorus_OTU418 |

**Table S6.** OTUs found in *Montipora digitata* eggs and adults. OTUs found in three or more *M. digitata* samples are in bold, less than three are not bolded. Egg values are averages including ± SE.

| **Dam** | **Life-stage,**  **N° OTUs** | **A OTUs (% ± SE)** | **C** | **D** | **B/*Ex-situ*/G/H/”uncultured”** |
| --- | --- | --- | --- | --- | --- |
| 7 | Adult, 7 | **A3.1_OTU5 (0.04),** A_2_OTU69 (0.039), **A_1_OTU4 (1.32)** | **C15_OTU1 (97.98)**, C1_8_OTU18 (0.53), **C15_2_OTU7 (0.039)** | **D1_OTU3 (0.039)** |  |
| 7 | Eggs  (n =11), 12 | **A3.1_OTU5 (0.08±0.05)** | **C15_9_OTU112 (0.003±0.002), C15_OTU1 (99.1±0.1)**,  **C1_4_OTU8 (0.04±0.02),**  **C15_3_OTU20 (0.001±0.0008), C15_6_OTU50 (0.002±0.001), C15_7_OTU102 (0.03±0.005), C15_5_OTU43* (0.004±0.003)** | **D1_OTU3 (0.2±0.05), D1a_OTU6 (0.02±0.02)** | Zoanthussociatus_1_OTU19 (0.02±0.02),  **Amphisorus_OTU2 (0.5±0.05)** |
| 8 | Adult, 5 | **A3.1_OTU5 (0.02),** **A_1_OTU4 (1.31)** | **C15_OTU1 (98.66)**, **C15_3_OTU20 (0.008)** | **D1_OTU3 (0.008)** |  |
| 8 | Eggs  (n = 12), 14 | **A3.1_OTU5 (0.17±0.06), A2_JCUSG-1_OTU32 (0.0006±0.0006)** | **C15_OTU1 (99.1±0.1)**,  **C15_9_OTU112 (0.0007±0.0007),**  C15_10_OTU117 (0.002±0.002),  **C1_4_OTU8 (0.02±0.01), C15_3_OTU20 (0.003±0.002), C15_6_OTU50 (0.001±0.001),**  C15_8_OTU103 (0.0004±0.0004), **C15_2_OTU7 (0.04±0.001), C15_7_OTU102 (0.0007±0.0007)** | **D1_OTU3 (0.3±0.1), D1a_OTU6 (0.04±0.03)** | **Amphisorus_OTU2 (0.5±0.04)** |
| 9 | Adult, 9 | **A3.1_OTU5 (0.009),** **A_1_OTU4 (0.32)** | **C15_OTU1 (98.8)**, **C1_4_OTU8 (0.097),** **C15_3_OTU20 (0.008)** | **D1_OTU3 (0.097), D1a_OTU6 (0.026)** | **Amphisorus_OTU2 (0.53),**  HA3-5 _OTU119 (0.017) |
| 9 | Eggs  (n = 12), 14 | **A3.1_OTU5 (0.07±0.04), CCMP828_OTU12 (0.07±0.04)** | **C15_OTU1 (98.4±0.1)**,  **C15_9_OTU112 (0.002±0.0009),**  C15_4_OTU33 (0.007±0.007),  **C1_4_OTU8 (0.034±0.01), C15_3_OTU20 (0.003±0.001), C15_6_OTU50 (0.002±0.001),**  C33_OTU82 (0.002±0.002), C15_8_OTU103 (0.0003±0.0003), **C15_2_OTU7 (0.03±0.01)** | **D1_OTU3 (0.3±0.1), D1a_OTU6 (0.1±0.05)** | **Amphisorus_OTU2 (0.6±0.06)** |
| 32 | Adult, 8 | **A_1_OTU4 (4.64),** A_2_OTU69 (0.045), | C1_7_OTU13 (0.009), **C15_OTU1 (95.2)**, **C15_3_OTU20 (0.009), C1_5_OTU10 (0.06),** **C15_2_OTU7 (0.027)** | **D1_OTU3 (0.009)** |  |
| 32 | Eggs  (n =5), 12 |  | **C15_OTU1 (99.3±0.4)**,  **C1_5_OTU10 (0.1±0.1),**  C1_9_OTU54 (0.01±0.01), **C15_9_OTU112 (0.002±0.002),** C1_7_OTU13 (0.2±0.2), **C15_7_OTU102 (0.002±0.002), C15_2_OTU7 (0.04±0.02) C1_4_OTU8 (0.01±0.01),**  **C15_3_OTU20 (0.002±0.002),** | D_OTU27 (0.04±0.04), **D1_OTU3 (0.2±0.1)** | Montiporafoliosa_OTU17 (0.03±0.03) |
| 28 | Adult, 2 | **A_1_OTU4 (0.02)** | **C15_OTU1 (99.97)** |  |  |
| 28 | Eggs  (n = 12), 14 | **A3.1_OTU5 (0.05±0.03),**  Microadriaticum_1__OTU29 (0.01±0.01), **CCMP828_OTU12 (0.03±0.02)** | **C15_OTU1 (99.7±0.05)**,  **C15_9_OTU112 (0.003±0.002), C1_4_OTU8 (0.007±0.006), C15_3_OTU20 (0.007±0.004), C15_6_OTU50 (0.0009±0.0009), C1_5_OTU10 (0.03±0.01), C15_7_OTU102 (0.001±0.001), C15_2_OTU7 (0.03±0.005)** | **D1_OTU3 (0.08±0.03), D1a_OTU6 (0.004±0.004)** | **Amphisorus_OTU2 (0.0006±0.00006),** Montiporafoliosa_OTU17 (0.007±0.07) |
| 26 | Adult, 7 | **A_1_OTU4 (0.009),**  A3.2_OTU68 (0.08), A2_OTU32 (0.25) | **C15_OTU1 (99.57), C15_6_OTU50 (0.009),**  **C1_5_OTU10 (0.045)** |  | G3.3_OTU67 (0.036) |
| 26 | Eggs  (n = 12), 12 | **A3.1_OTU5 (0.04±0.02), CCMP828_OTU12 (0.1±0.05)** | **C15_OTU1 (99.4±0.1)**,  **C15_9_OTU112 (0.0009±0.0006), C15_3_OTU20 (0.008±0.003), C_1_OTU47 (0.001±0.001), C15_6_OTU50 (0.004±0.004), C1_5_OTU10 (0.1±0.05), C15_2_OTU7 (0.02±0.01)** | **D1_OTU3 (0.24±0.07), D1a_OTU6 (0.03±0.02)** | “uncultured”_OTU59 (0.02±0.02) |
| 24 | Adult, 2 |  | **C15_OTU1 (99.53)** |  | **Amphisorus_OTU2 (0.47)** |
| 24 | Eggs  (n = 12), 13 | **A3.1_OTU5 (0.07±0.06), CCMP828_OTU12 (0.02±0.02),**  **A_1_OTU4 (0.0004±0.0004)** | **C15_OTU1 (99.2±0.1)**,  **C15_9_OTU112 (0.002±0.002), C1_4_OTU8 (0.02±0.007), C15_3_OTU20 (0.003±0.001), C15_6_OTU50 (0.004±0.002), C15_2_OTU7 (0.05±0.02), C15_5_OTU43* (0.004±0.002)** | **D1_OTU3 (0.2±0.06), D1a_OTU6 (0.01±0.01)** | **Amphisorus_OTU2 (0.4±0.04)** |
| 11 | Adult, 5 | **CCMP828_OTU12 (0.035)** | **C15_OTU1 (99.87), C1_4_OTU8 (0.046), C15_2_OTU7 (0.01)** | **D1_OTU3 (0.034)** |  |
| 11 | Eggs  (n = 11), 12 | **A3.1_OTU5 (0.18±0.1), CCMP828_OTU12 (0.05±0.03)** | **C15_OTU1 (99.6±0.09)**, **C15_9_OTU112 (0.002±0.001), C1_4_OTU8 (0.05±0.02), C15_3_OTU20 (0.004±0.002), C15_6_OTU50 (0.003±0.001),** C1_16_OTU104 (0.005±0.005), **C15_2_OTU7 (0.02±0.007)** | **D1_OTU3 (0.06±0.02), D1a_OTU6 (0.015±0.01)** | B1_OTU31 (.007±0.007) |
| 29 | Adult, 3 | **A_1_OTU4 (0.73)** | **C15_OTU1 (99.3)** |  | **Amphisorus_OTU2 (0.008)** |
| 29 | Eggs  (n =12), 77 | **A2_meandrinae.1_OTU56 (0.009±0.006),**  A2_meandrinae.2_OTU90 (0.004±0.004),  **A3.1_OTU5 (0.5±0.3),**  A2_meandrinae.3_OTU113 (0.004±0.003),  **Asand_Oku17_OTU111 (0.004±0.003),**  CCMP2455_OTU110 (0.003±0.003),  **CCMP828_OTU12 (0.02±0.02)** | **C15_9_OTU112 (0.001±0.0007),**  **C15_1_OTU1 (97.7±0.9),**  **C15_3_OTU20** (0.002±0.001),  **C15_6_OTU50 (0.002±0.002),**  **C1_5_OTU10 (0.009±0.006),**  **C15_2_OTU7 (0.03±0.01),** | **D1_OTU3 (0.5±0.4),**  **D1a_OTU6 (0.03±0.03)** | **Amphisorus_OTU2 (0.001±0.001),** “uncultured”34_OTU78 (0.004±0.004),  **“uncultured”5_OTU23 (0.04±0.02),**  **“uncultured”16_OTU42 (0.015±0.01),**  “uncultured”56_OTU130 (0.004±0.003),  “uncultured”41_OTU98 (0.009±0.008),  “uncultured”58_OTU133 (0.004±0.003),  **“uncultured”31_OTU74 (0.01±0.006),**  **“uncultured”21_OTU53 (0.005±0.003),**  **“uncultured”14_OTU40 (0.016±0.01),**  **“uncultured”33_OTU76 (0.01±0.007),**  **“uncultured”20_OTU52 (0.02±0.01),**  **“uncultured”11_OTU37 (0.02±0.01),**  **“uncultured”23_OTU57 (0.02±0.01),**  **“uncultured”18_OTU46 (0.02±0.01),**  **“uncultured”10_OTU35 (0.02±0.01),**  “uncultured”32_OTU75 (0.01±0.008),  “uncultured”48_OTU120 (0.004±0.004),  “uncultured”45_OTU107 (0.003±0.002),  **“uncultured”4_OTU21 (0.03±0.02),**  **“uncultured”9_OTU34 (0.03±0.02),**  **“uncultured”6_OTU26 (0.02±0.01),**  “uncultured”47_OTU116 (0.004±0.003),  “uncultured”19_OTU49 (0.008±0.006),  “uncultured”49_OTU121 (0.004±0.004),  **“uncultured”35_OTU80 (0.006±0.004),**  “uncultured”44_OTU101 (0.005±0.004),  “uncultured”60_OTU135 (0.003±0.003),  **“uncultured”26_OTU65 (0.006±0.004),**  **“uncultured”30_OTU73 (0.01±0.008),**  “uncultured”55_OTU129 (0.006±0.005),  **“uncultured”28_OTU71 (0.014±0.01),**  **“uncultured”61_OTU136 (0.003±0.002),**  **“uncultured”27_OTU70 (0.007±0.004),**  **“uncultured”29_OTU72 (0.007±0.004),**  “uncultured”42_OTU99 (0.003±0.002),  **“uncultured”36_OTU83 (0.005±0.003),**  **“uncultured”17_OTU45 (0.006±0.004),**  **“uncultured”13_OTU39 (0.02±0.01),**  “uncultured”38_OTU93 (0.003±0.002),  “uncultured”43_OTU100 (0.004±0.003),  “uncultured”39_OTU94 (0.004±0.003),  **“uncultured”54_OTU128 (0.008±0.01),**  **“uncultured”37_OTU85 (0.005±0.003),**  “uncultured”50_OTU122 (0.004±0.003),  “uncultured”40_OTU97 (0.003±0.003),  “uncultured”57_OTU132 (0.002±0.001),  “uncultured”59_OTU134 (0.009±0.01),  **“uncultured”1_OTU9 (0.4±0.2),**  **“uncultured”52_OTU126 (0.005±0.003),**  **“uncultured”25_OTU61 (0.007±0.004),**  **“uncultured”8_OTU30 (0.03±0.02),**  **“uncultured”22_OTU55 (0.02±0.01),**  **“uncultured”12_OTU38 (0.01±0.006),**  **“uncultured”15_OTU41 (0.01±0.008),**  **“uncultured”3_OTU15 (0.08±0.04),**  “uncultured”51_OTU123 (0.003±0.003),  **“uncultured”2_OTU14 (0.1±0.06),**  “uncultured”46_OTU109 (0.005±0.01),  **“uncultured”24_OTU60 (0.007±0.003),**  **“uncultured”7_OTU28 (0.02±0.01),**  “uncultured”53_OTU127 (0.003±0.003) |

**Table S7.** DESeq2 comparisons of different egg families. P-adj values represent DESeq2 Bejamini-Hochberg p-adjusted values for multiple comparisons.

| **Dam identity** | **Dam identity** | **OTU name** | **Padj** |
| --- | --- | --- | --- |
| 26 | 28 | OTU_10_C1_5 | 9.5e-03 |
| 26 | 8 | OTU_10_C1_5 | 9.5e-03 |
| 26 | 9 | OTU_10_C1_5 | 1.6e-02 |
| 24 | 26 | OTU_10_C1_5 | 1.e-02 |
| 29 | 7 | OTU_14_”uncultured”2 | 3.2e-02 |
| 29 | 8 | OTU_14_”uncultured”2 | 3.3e-02 |
| 29 | 9 | OTU_14_”uncultured”2 | 3.3e-02 |
| 24 | 29 | OTU_14_”uncultured”2 | 3.1e-02 |
| 26 | 29 | OTU_14_”uncultured”2 | 0.03 |
| 11 | 29 | OTU_14_”uncultured”2 | 0.04 |
| 28 | 29 | OTU_14_”uncultured”2 | 0.05 |
| 24 | 28 | OTU_2_Amphisorushemprichii | 1.8e-13 |
| 24 | 32 | OTU_2_Amphisorushemprichii | 3.4e-08 |
| 32 | 7 | OTU_2_Amphisorushemprichii | 2.2e-08 |
| 11 | 24 | OTU_2_Amphisorushemprichii | 1.4e-12 |
| 11 | 9 | OTU_2_Amphisorushemprichii | 1.8e-13 |
| 28 | 8 | OTU_2_Amphisorushemprichii | 3.8e-13 |
| 28 | 9 | OTU_2_Amphisorushemprichii | 3.8e-13 |
| 32 | 8 | OTU_2_Amphisorushemprichii | 5.6e-08 |
| 11 | 8 | OTU_2_Amphisorushemprichii | 3e-12 |
| 32 | 9 | OTU_2_Amphisorushemprichii | 8e-09 |
| 24 | 26 | OTU_2_Amphisorushemprichii | 1.3e-12 |
| 26 | 28 | OTU_2_Amphisorushemprichii | 2.3e-12 |
| 26 | 8 | OTU_2_Amphisorushemprichii | 2.3e-12 |
| 26 | 9 | OTU_2_Amphisorushemprichii | 4.1e-13 |
| 24 | 29 | OTU_2_Amphisorushemprichii | 3.6e-14 |
| 29 | 8 | OTU_2_Amphisorushemprichii | 8.6e-14 |
| 29 | 9 | OTU_2_Amphisorushemprichii | 8.6e-14 |
| 29 | 7 | OTU_2_Amphisorushemprichii | 2.1e-14 |
| 24 | 29 | OTU_8_C1_4 | 3.1e-02 |
| 29 | 8 | OTU_8_C1_4 | 3.3e-02 |
| 29 | 9 | OTU_8_C1_4 | 3.3e-02 |
| 29 | 7 | OTU_8_C1_4 | 1.4e-02 |
| 11 | 29 | OTU_8_C1_4 | 0.01 |
| 29 | 7 | OTU_9_”uncultured”1 | 3.4e-02 |
| 29 | 8 | OTU_9_”uncultured”1 | 3.4e-02 |
| 29 | 9 | OTU_9_”uncultured”1 | 3.4e-02 |
| 24 | 29 | OTU_9_”uncultured”1 | 3.4e-02 |
| 11 | 29 | OTU_9_”uncultured”1 | 0.04 |
| 26 | 29 | OTU_9_”uncultured”1 | 0.05 |
| 28 | 29 | OTU_9_”uncultured”1 | 0.05 |

**Table S8.** Full taxonomic designations for OTUs on Figures 2 and 4. OTUs shared between the two *A. tenuis* years. Red OTUs in the table represent those where NCBI Blast identification did not match dendrogram clustering positions.

| Dataset | Shared OTUs | OTU | Id Neighbour Joining clustering | NCBI Blast id | Shared OTUs number |
| --- | --- | --- | --- | --- | --- |
| *2012_A.tenuis* |  | 30 | C | D |  |
| *2012_A.tenuis* |  | 235 | C | D |  |
| *2012_A.tenuis* |  | 170 | C | D |  |
| *2012_A.tenuis* |  | 57 | C | 12979_F2? |  |
| *2012_A.tenuis* |  | 150 | C | D |  |
| *2012_A.tenuis* |  | 160 | C | C_PtMil1-9 |  |
| *2012_A.tenuis* |  | 270 | C | OTU18 |  |
| *2012_A.tenuis* |  | 189 | C | C3 |  |
| *2012_A.tenuis* |  | 67 | C | A |  |
| *2012_A.tenuis* |  | 123 | C | C_SC13.7 |  |
| *2012_A.tenuis* | * | 194 | C | C3 | 347 |
| *2012_A.tenuis* |  | 37 | A1 | A1 |  |
| *2012_A.tenuis* |  | 188 | A1 | A1 |  |
| *2012_A.tenuis* |  | 103 | A1 | A1 |  |
| *2012_A.tenuis* |  | 118 | A1 | A1 |  |
| *2012_A.tenuis* |  | 14 | A1 | A1 |  |
| *2012_A.tenuis* |  | 4 | A1 | A1 |  |
| *2012_A.tenuis* |  | 23 | A1 | A1 |  |
| *2012_A.tenuis* |  | 323 | A1 | A1 |  |
| *2012_A.tenuis* |  | 128 | C1 | C1 |  |
| *2012_A.tenuis* |  | 274 | C1 | 13478_F2 |  |
| *2012_A.tenuis* |  | 249 | C | C1v18 |  |
| *2012_A.tenuis* |  | 354 | C1 | C1 |  |
| *2012_A.tenuis* | * | 81 | G | A3 | 159 |
| *2012_A.tenuis* |  | 119 | G | A3 |  |
| *2012_A.tenuis* |  | 24 | G2 | G2 |  |
| *2012_A.tenuis* | * | 6 | C | D | 101 |
| *2012_A.tenuis* | * | 10 | C | D | 10 |
| *2012_A.tenuis* |  | 46 | C | D |  |
| *2012_A.tenuis* | * | 15 | C | “uncultured” | 33 |
| *2012_A.tenuis* | * | 38 | C | “uncultured” | 97 |
| *2012_A.tenuis* |  | 139 | C | *Zoanthus* |  |
| *2012_A.tenuis* | * | 75 | C | “uncultured” | 180 |
| *2012_A.tenuis* |  | 259 | C | OTU18 |  |
| *2012_A.tenuis* |  | 346 | C1 | C1 |  |
| *2012_A.tenuis* | * | 121 | C1 | C1 | 113 |
| *2012_A.tenuis* |  | 269 | C1 | C1 |  |
| *2012_A.tenuis* |  | 107 | C1 | C1 |  |
| *2012_A.tenuis* |  | 45 | C1 | C1 |  |
| *2012_A.tenuis* |  | 83 | C1 | C1 |  |
| *2012_A.tenuis* | * | 64 | C | CCMP2455_F2 | 50 |
| *2012_A.tenuis* |  | 209 | C | *Amphisorus* |  |
| *2012_A.tenuis* | * | 109 | C, probably F | *Amphisorus* | 418 |
| *2012_A.tenuis* |  | 340 | C1 | C1 |  |
| *2012_A.tenuis* | * | 54 | C or G | D1 | 361 |
| *2012_A.tenuis* |  | 12 | C |  |  |
| *2012_A.tenuis* |  | 19 | C90 |  |  |
| *2012_A.tenuis* | * | 4 | C1 |  | 1 |
| *2012_A.tenuis* | * | 8 | C | “uncultured” | 104 |
| *2012_A.tenuis* |  | 31 | C | *Favia* |  |
| *2012_A.tenuis* |  | 520 | C | *Amphisours* |  |
| *2012_A.tenuis* |  | 455 | C15 |  |  |
| *2012_A.tenuis* |  | 61 | C |  |  |
| *2012_A.tenuis* |  | 513 | C90 |  |  |
| *2012_A.tenuis* | * | 91 | C |  | 232 |
| *2012_A.tenuis* | * | 58 | C | 1635 | 13 |
| *2012_A.tenuis* | * | 9 | G4 | G4 | 68 |
| *2012_A.tenuis* |  | 16 | G4 | G4 |  |
| *2012_A.tenuis* |  | 96 | D1a |  |  |
| *2012_A.tenuis* |  | 295 | D1 |  |  |
| *2012_A.tenuis* | * | 146 | D1a |  | 142 |
| *2012_A.tenuis* |  | 461 | D1a |  |  |
| *2012_A.tenuis* |  | 337 | D1 |  |  |
| *2012_A.tenuis* |  | 363 | D1 |  |  |
| *2012_A.tenuis* | * | 1 | D1 |  | 2 |
| *2012_A.tenuis* |  | 369 | D1 |  |  |
| *2012_A.tenuis* |  | 430 | D1a |  |  |
| *2012_A.tenuis* |  | 486 | D1a |  |  |
| *2012_A.tenuis* |  | 5 | D1a |  |  |
| *2012_A.tenuis* | * | 87 | D | C | 137 |
| *2012_A.tenuis* | * | 106 | D | “uncultured” | 12 |
| *2012_A.tenuis* | * | 51 | B1 | B1 | 45 |
| *2012_A.tenuis* |  | 117 | C | C |  |
| *2012_A.tenuis* |  | 104 | C | 1679 |  |
| *2012_A.tenuis* |  | 237 | A | 1679 |  |
| *2012_A.tenuis* | * | 22 | A | mizugama | 91 |
| *2012_A.tenuis* | * | 65 | A4 | A4 | 114 |
| *2012_A.tenuis* | * | 18 | A | B4 | 17 |
| *2012_A.tenuis* |  | 351 | A3 | A3 |  |
| *2012_A.tenuis* | * | 526 | A3 | A3 | 665 |
| *2012_A.tenuis* | * | 3 | A3 | A3 | 3 |
| *2012_A.tenuis* |  | 314 | A3 | A1_CCMP828 |  |
| *2012_A.tenuis* | * | 174 | A3 | A3 | 491 |
| *2012_A.tenuis* |  | 166 | A | Oga03 |  |
| *2012_A.tenuis* | * | 56 | A1 | A1_CCMP828 | 458 |
| *2012_A.tenuis* |  | 505 | A | A1_CCMP828 |  |
| *2012_A.tenuis* |  | 319 | A1_3B-J |  |  |
| *2012_A.tenuis* |  | 395 | A | A1_CCMP828 |  |
| *2012_A.tenuis* | * | 2 | A | A1_CCMP828 | 7 |
| *2012_A.tenuis* |  | 394 | A13 | A13 |  |
| *2012_A.tenuis* |  | 412 | A | D1a |  |
| *2012_A.tenuis* | * | 29 | A13 | A13 | 57 |
| *2012_A.tenuis* | * | 132 | A | A1_CCMP828 | 664 |
| *2012_A.tenuis* | * | 39 | A2 | A2_JCUSG-1 | 66 |
| *2012_A.tenuis* | * | 59 | A | *Zoanthus* | 352 |
| *2012_A.tenuis* |  | 519 | A | C |  |
| *2012_A.tenuis* | * | 148 | A | *Zoanthus* | 117 |
| *2012_A.tenuis* | * | 449 | A | *Zoanthus* | 178 |
| *2012_A.tenuis* |  | 80 | A | *Zoanthus* |  |
| *2012_A.tenuis* |  | 13 | A | *Zoanthus* |  |
| *2012_A.tenuis* | * | 540 | A | *Zoanthus* | 49 |
| *2012_A.tenuis* | * | 41 | A | *Zoanthus* | 28 |
| *2012_A.tenuis* |  | 93 | A | *Zoanthus* |  |
| *2012_A.tenuis* |  | 127 | A | *Zoanthus* |  |
| *2012_A.tenuis* | * | 11 | A | *Zoanthus* | 21 |
| *2012_A.tenuis* |  | 20 | A | zoox23 |  |
| *2012_A.tenuis* |  | 129 | A | Scyphozoan |  |
| *2012_A.tenuis* | * | 28 | A | Scyphozoan | 120 |
| *2012_A.tenuis* | * | 33 | A | A | 69 |
| *2012_A.tenuis* | * | 23 | A | Scyphozoan | 34 |
| *2012_A.tenuis* | * | 47 | A | Scyphozoan | 84 |
| *2012_A.tenuis* | * | 7 | A | “uncultured” | 43 |
| *2012_A.tenuis* |  | 32 | A | A_mami1i |  |
| *2012_A.tenuis* |  | 210 | A | A_HI-0609 |  |
| *2012_A.tenuis* | * | 68 | A3 | A3 | 238 |
| *2012_A.tenuis* |  | 102 | A3 | A3 |  |
| *2012_A.tenuis* | * | 17 | A | zoox21 | 35 |
| *2012_A.tenuis* | * | 176 | A | zoox23 | 186 |
| *2012_A.tenuis* |  | 21 | A | zoox23 |  |
| *2012_A.tenuis* |  | 50 | A | zoox23 |  |
| *2012_A.tenuis* | * | 35 | A | “uncultured” | 103 |
| *2012_A.tenuis* | * | 135 | A | “uncultured” | 129 |
| *2012_A.tenuis* |  | 142 | C | “uncultured” |  |
| *2012_A.tenuis* | * | 14 | C | Zoanthus | 32 |
| *2012_A.tenuis* |  | 342 | C1 | C1 |  |
| *2012_A.tenuis* | * | 347 | C1 | C | 140 |
| *2012_A.tenuis* |  | 55 | C1 | B2 |  |
| *2012_A.tenuis* | * | 105 | C1 | C1 | 492 |
| *2012_A.tenuis* |  | 206 | C1 | C1 |  |
| *2012_A.tenuis* | * | 216 | C1 | C1 | 381 |
| *2012_A.tenuis* | * | 223 | C1 | C1 | 249 |
| *2012_A.tenuis* |  | 275 | C1 | C1 |  |
| *2012_A.tenuis* |  | 53 | C1 | zoox23 |  |
| *2012_A.tenuis* | * | 173 | C1 | zoox23 | 227 |
| *2013_A.tenuis* |  | 83 | A | “uncultured” |  |
| *2013_A.tenuis* |  | 99 | *S.natans*(A_III_) | *S.natans*(A_III_) |  |
| *2013_A.tenuis* | * | 34 | A | *Amphisorus* | 23 |
| *2013_A.tenuis* | * | 84 | A | 1Sy24 | 47 |
| *2013_A.tenuis* | * | 120 | A | 1Sy24 | 28 |
| *2013_A.tenuis* |  | 39 | A | 1Sy24 |  |
| *2013_A.tenuis* |  | 41 | A | 1Sy24 |  |
| *2013_A.tenuis* |  | 40 | A | “uncultured” |  |
| *2013_A.tenuis* | * | 69 | A | A | 33 |
| *2013_A.tenuis* |  | 604 | A | A |  |
| *2013_A.tenuis* |  | 198 | A | “uncultured” |  |
| *2013_A.tenuis* |  | 468 | A | “uncultured” |  |
| *2013_A.tenuis* | * | 33 | C | “uncultured” | 15 |
| *2013_A.tenuis* | * | 97 | C | “uncultured” | 38 |
| *2013_A.tenuis* | * | 180 | C | “uncultured” | 75 |
| *2013_A.tenuis* |  | 42 | G | “uncultured” |  |
| *2013_A.tenuis* |  | 55 | G | “uncultured” |  |
| *2013_A.tenuis* |  | 130 | G | “uncultured” |  |
| *2013_A.tenuis* |  | 47 | G6 | G6 |  |
| *2013_A.tenuis* |  | 61 | G | F_1679 |  |
| *2013_A.tenuis* |  | 70 | G | *S. voratum* (E) |  |
| *2013_A.tenuis* |  | 94 | G2b | G2b |  |
| *2013_A.tenuis* |  | 169 | G2b | G2b |  |
| *2013_A.tenuis* | * | 159 | G | A3 | 81 |
| *2013_A.tenuis* | * | 68 | G4 | G4 | 9 |
| *2013_A.tenuis* | * | 361 | G or C | D1.2 | 54 |
| *2013_A.tenuis* |  | 8 | G6 | G6 |  |
| *2013_A.tenuis* |  | 48 | G3.4 | G3.4 |  |
| *2013_A.tenuis* | * | 1 | C1 | C1 |  |
| *2013_A.tenuis* | * | 104 | C1 | “uncultured” | 8 |
| *2013_A.tenuis* |  | 716 | C1v1e | C1v1e |  |
| *2013_A.tenuis* |  | 63 | C | C |  |
| *2013_A.tenuis* |  | 661 | C1 | C1 |  |
| *2013_A.tenuis* |  | 567 | C1 | C1 |  |
| *2013_A.tenuis* |  | 762 | C1 | C1 |  |
| *2013_A.tenuis* |  | 800 | C | C |  |
| *2013_A.tenuis* |  | 124 | C | 1635_Fr3? |  |
| *2013_A.tenuis* |  | 157 | C | 1635_Fr3? |  |
| *2013_A.tenuis* | * | 13 | C | 1635_Fr3? | 58 |
| *2013_A.tenuis* | * | 232 | C |  | 91 |
| *2013_A.tenuis* |  | 14 | F1 |  |  |
| *2013_A.tenuis* |  | 163 | F1 | CCMP2455_F2 |  |
| *2013_A.tenuis* | * | 50 | F1 | CCMP2455_F2 | 64 |
| *2013_A.tenuis* |  | 81 | F5 | 1363_F5 |  |
| *2013_A.tenuis* |  | 353 | F5 | 1363_F5 |  |
| *2013_A.tenuis* |  | 106 | F5 | 13467_F5 |  |
| *2013_A.tenuis* |  | 93 | F4.5 |  |  |
| *2013_A.tenuis* | * | 418 | F4.5 | *Amphisorus* | 109 |
| *2013_A.tenuis* |  | 803 | F4.5 | C1 |  |
| *2013_A.tenuis* |  | 121 | F4.5 | C1 |  |
| *2013_A.tenuis* |  | 119 | F4.5 | 1631 |  |
| *2013_A.tenuis* |  | 96 | F3.2 | F3.2 |  |
| *2013_A.tenuis* |  | 134 | F3.2 | *Amphisorus* |  |
| *2013_A.tenuis* |  | 102 | F3.2 | 1681 |  |
| *2013_A.tenuis* | * | 45 | B1 | B1 | 51 |
| *2013_A.tenuis* |  | 6 | D1a | D1a |  |
| *2013_A.tenuis* |  | 727 | D1a | D1a |  |
| *2013_A.tenuis* |  | 331 | D | D |  |
| *2013_A.tenuis* | * | 2 | D1 | D1 | 1 |
| *2013_A.tenuis* |  | 737 | D1a | D1a |  |
| *2013_A.tenuis* | * | 142 | D1a | D1a | 146 |
| *2013_A.tenuis* |  | 678 | D | D |  |
| *2013_A.tenuis* |  | 756 | D1a | D1a |  |
| *2013_A.tenuis* | * | 12 | D | “uncultured” | 106 |
| *2013_A.tenuis* | * | 137 | D |  | 87 |
| *2013_A.tenuis* |  | 145 | D |  |  |
| *2013_A.tenuis* |  | 440 | C1 |  |  |
| *2013_A.tenuis* |  | 237 | C1 |  |  |
| *2013_A.tenuis* | * | 113 | C1 |  | 121 |
| *2013_A.tenuis* |  | 633 | C1 |  |  |
| *2013_A.tenuis* |  | 60 | C | “uncultured” |  |
| *2013_A.tenuis* |  | 731 | C | zoox23 |  |
| *2013_A.tenuis* |  | 67 | C | *Amphisorus* |  |
| *2013_A.tenuis* | * | 347 | C3 |  | 194 |
| *2013_A.tenuis* | * | 10 | C | D | 10 |
| *2013_A.tenuis* | * | 101 | C | D | 6 |
| *2013_A.tenuis* |  | 16 | C | D |  |
| *2013_A.tenuis* |  | 4 | C | D |  |
| *2013_A.tenuis* |  | 5 | C | D |  |
| *2013_A.tenuis* |  | 58 | C | D |  |
| *2013_A.tenuis* | * | 227 | C | zoox23 | 173 |
| *2013_A.tenuis* |  | 392 | C | zoox23 |  |
| *2013_A.tenuis* |  | 79 | C1 |  |  |
| *2013_A.tenuis* |  | 86 | C1 |  |  |
| *2013_A.tenuis* | * | 249 | C1 |  | 223 |
| *2013_A.tenuis* | * | 381 | C1 |  | 216 |
| *2013_A.tenuis* |  | 374 | C | D |  |
| *2013_A.tenuis* |  | 426 | C | D |  |
| *2013_A.tenuis* |  | 373 | C | D |  |
| *2013_A.tenuis* | * | 492 | C1 |  | 105 |
| *2013_A.tenuis* |  | 287 | C1 |  |  |
| *2013_A.tenuis* |  | 423 | C1 |  |  |
| *2013_A.tenuis* |  | 439 | C |  |  |
| *2013_A.tenuis* |  | 593 | C | D1 |  |
| *2013_A.tenuis* |  | 181 | C | “uncultured” |  |
| *2013_A.tenuis* |  | 659 | C |  |  |
| *2013_A.tenuis* |  | 37 | C | D |  |
| *2013_A.tenuis* |  | 409 | C | “uncultured” |  |
| *2013_A.tenuis* |  | 164 | C | “uncultured” |  |
| *2013_A.tenuis* |  | 430 | C | “uncultured” |  |
| *2013_A.tenuis* |  | 76 | C_SC13.7 |  |  |
| *2013_A.tenuis* |  | 514 | C | “uncultured” |  |
| *2013_A.tenuis* |  | 382 | C | “uncultured” |  |
| *2013_A.tenuis* |  | 432 | C1 | C1 |  |
| *2013_A.tenuis* |  | 22 | C | 1Sy23 |  |
| *2013_A.tenuis* |  | 73 | C | CCMP2456 |  |
| *2013_A.tenuis* |  | 558 | C | CCMP2456 |  |
| *2013_A.tenuis* |  | 457 | C | CCMP2456 |  |
| *2013_A.tenuis* |  | 80 | C1 | C1 |  |
| *2013_A.tenuis* |  | 311 | C | 1Sy23 |  |
| *2013_A.tenuis* |  | 51 | C | *Amphisorus* |  |
| *2013_A.tenuis* |  | 31 | C | “uncultured” |  |
| *2013_A.tenuis* | * | 32 | C | *Zoanthus* |  |
| *2013_A.tenuis* | * | 140 | C | C |  |
| *2013_A.tenuis* | * | 352 | A | *Zoanthus* | 59 |
| *2013_A.tenuis* | * | 28 | A | *Zoanthus* | 41 |
| *2013_A.tenuis* | * | 49 | A | *Zoanthus* | 540 |
| *2013_A.tenuis* |  | 224 | A | *Zoanthus* |  |
| *2013_A.tenuis* | * | 117 | A | *Zoanthus* | 148 |
| *2013_A.tenuis* | * | 178 | A | *Zoanthus* | 449 |
| *2013_A.tenuis* |  | 18 | A | *Zoanthus* |  |
| *2013_A.tenuis* |  | 26 | A | *Zoanthus* |  |
| *2013_A.tenuis* |  | 88 | A | *Zoanthus* |  |
| *2013_A.tenuis* |  | 144 | A | *Zoanthus* |  |
| *2013_A.tenuis* | * | 21 | A | *Zoanthus* | 11 |
| *2013_A.tenuis* |  | 316 | A | *Zoanthus* |  |
| *2013_A.tenuis* |  | 24 | A | A_HI-0609 |  |
| *2013_A.tenuis* |  | 27 | A | “uncultured” |  |
| *2013_A.tenuis* | * | 664 | A | CCMP828_A1 | 132 |
| *2013_A.tenuis* | * | 57 | A13 | A13 | 29 |
| *2013_A.tenuis* | * | 66 | A_JCUSG-1 | A_JCUSG-1 | 39 |
| *2013_A.tenuis* | * | 3 | A3 | A3 | 3 |
| *2013_A.tenuis* |  | 799 | A3 | A3 |  |
| *2013_A.tenuis* | * | 665 | A3 | A3 | 526 |
| *2013_A.tenuis* |  | 725 | A3 | A3 |  |
| *2013_A.tenuis* | * | 491 | A3 | A3 | 174 |
| *2013_A.tenuis* |  | 30 | A | “uncultured” |  |
| *2013_A.tenuis* | * | 7 | CCMP828_A1 | CCMP828_A1 | 2 |
| *2013_A.tenuis* |  | 746 | CCMP828_A1 | CCMP828_A1 |  |
| *2013_A.tenuis* |  | 476 | A3 | A3 |  |
| *2013_A.tenuis* | * | 458 | CCMP828_A1 | CCMP828_A1 | 56 |
| *2013_A.tenuis* |  | 408 | A3 | A3 |  |
| *2013_A.tenuis* | * | 129 | A | “uncultured” | 135 |
| *2013_A.tenuis* |  | 333 | A | 1Sy23 |  |
| *2013_A.tenuis* | * | 103 | A | “uncultured” | 35 |
| *2013_A.tenuis* |  | 98 | A | 1679 |  |
| *2013_A.tenuis* |  | 174 | A | 1679 |  |
| *2013_A.tenuis* | * | 91 | A | mizugama | 22 |
| *2013_A.tenuis* | * | 114 | A4.3 |  | 65 |
| *2013_A.tenuis* |  | 133 | A | *S. muscatinei* (B4) |  |
| *2013_A.tenuis* |  | 109 | A_HI-0509 |  |  |
| *2013_A.tenuis* |  | 139 | A_HI-0509 |  |  |
| *2013_A.tenuis* |  | 334 | A_HI-0509 |  |  |
| *2013_A.tenuis* | * | 17 | A | *S. muscatinei* (B4) | 18 |
| *2013_A.tenuis* |  | 118 | A_HI-0509 |  |  |
| *2013_A.tenuis* |  | 85 | A | zoox23 |  |
| *2013_A.tenuis* |  | 291 | A3 | A3 |  |
| *2013_A.tenuis* | * | 238 | A3 | A3 | 68 |
| *2013_A.tenuis* |  | 309 | A3 | A3 |  |
| *2013_A.tenuis* |  | 77 | A3 | A3 |  |
| *2013_A.tenuis* | * | 43 | A | “uncultured” | 7 |
| *2013_A.tenuis* |  | 9 | A | zoox23 |  |
| *2013_A.tenuis* |  | 46 | A | zoox23 |  |
| *2013_A.tenuis* |  | 54 | A | zoox23 |  |
| *2013_A.tenuis* |  | 195 | A | zoox23 |  |
| *2013_A.tenuis* |  | 342 | A | zoox23 |  |
| *2013_A.tenuis* |  | 19 | A | zoox23 |  |
| *2013_A.tenuis* |  | 403 | A | zoox23 |  |
| *2013_A.tenuis* |  | 23 | A | C1 |  |
| *2013_A.tenuis* |  | 59 | A | zoox23 |  |
| *2013_A.tenuis* |  | 443 | A | zoox23 |  |
| *2013_A.tenuis* |  | 156 | A | zoox23 |  |
| *2013_A.tenuis* |  | 750 | A | zoox23 |  |
| *2013_A.tenuis* |  | 56 | A | zoox23 |  |
| *2013_A.tenuis* | * | 186 | A | zoox23 | 176 |
| *2013_A.tenuis* | * | 35 | A | zoox21 | 17 |
| *2013_A.tenuis* |  | 52 | A | zoox23 |  |
| *2013_A.tenuis* |  | 592 | A | zoox23 |  |
| *2013_A.tenuis* |  | 170 | A | zoox23 |  |
| *2013_A.tenuis* |  | 11 | A13 | A13 |  |
| *2013_A.tenuis* |  | 177 | A | “uncultured” |  |
| *M. digitata* |  | 65 | A |  |  |
| *M. digitata* |  | 121 | A |  |  |
| *M. digitata* |  | 127 | A |  |  |
| *M. digitata* |  | 113 | A2 |  |  |
| *M. digitata* |  | 123 | A |  |  |
| *M. digitata* |  | 134 | A2 |  |  |
| *M. digitata* |  | 34 | A13 |  |  |
| *M. digitata* |  | 42 | C | “uncultured”/*S. voratum* |  |
| *M. digitata* |  | 102 | C15 |  |  |
| *M. digitata* |  | 1 | C15 |  |  |
| *M. digitata* |  | 120 | C | Uncutlured/A1 |  |
| *M. digitata* |  | 130 | C |  |  |
| *M. digitata* |  | 94 | C |  |  |
| *M. digitata* |  | 101 | C |  |  |
| *M. digitata* |  | 128 | C | Uncutlured/A1 |  |
| *M. digitata* |  | 116 | C1 |  |  |
| *M. digitata* |  | 9 | A2 |  |  |
| *M. digitata* |  | 93 | A | “uncultured”/*S. voratum* |  |
| *M. digitata* |  | 129 | A |  |  |
| *M. digitata* |  | 133 | A |  |  |
| *M. digitata* |  | 23 | A | “uncultured”/*S. voratum* |  |
| *M. digitata* |  | 111 | A |  |  |
| *M. digitata* |  | 135 | F |  |  |
| *M. digitata* |  | 100 | F |  |  |
| *M. digitata* |  | 110 | F |  |  |
| *M. digitata* |  | 10 | C1 |  |  |
| *M. digitata* |  | 60 | D1a | “uncultured”/A1 |  |
| *M. digitata* |  | 14 | D1a | “uncultured”/C1 |  |
| *M. digitata* |  | 6 | D1a |  |  |
| *M. digitata* |  | 12 | A |  |  |
| *M. digitata* |  | 30 | C1 |  |  |
| *M. digitata* |  | 122 | C |  |  |
| *M. digitata* |  | 132 | C |  |  |
| *M. digitata* |  | 52 | C15 |  |  |
| *M. digitata* |  | 53 | C | “uncultured”/A |  |
| *M. digitata* |  | 117 | C15 |  |  |
| *M. digitata* |  | 97 | C |  |  |
| *M. digitata* |  | 85 | C1 |  |  |
| *M. digitata* |  | 99 | C1 | *S. voratum* |  |
| *M. digitata* |  | 136 | C |  |  |
| *M. digitata* |  | 78 | C1 |  |  |
| *M. digitata* |  | 80 | C |  |  |
| *M. digitata* |  | 98 | C1 | “uncultured”/A |  |
| *M. digitata* |  | 109 | C |  |  |
| *M. digitata* |  | 83 | C |  |  |
| *M. digitata* |  | 107 | C1 | *S. voratum* |  |
| *M. digitata* |  | 126 | C |  |  |
| *M. digitata* |  | 46 | C1 |  |  |
| *M. digitata* |  | 7 | C15 |  |  |
| *M. digitata* |  | 18 | C1 |  |  |
| *M. digitata* |  | 19 | A3 |  |  |
| *M. digitata* |  | 27 | D |  |  |
| *M. digitata* |  | 15 | C1 |  |  |
| *M. digitata* |  | 20 | C15 |  |  |
| *M. digitata* |  | 17 | C15 |  |  |
| *M. digitata* |  | 26 | C1 |  |  |
| *M. digitata* |  | 29 | A1 |  |  |
| *M. digitata* |  | 59 | A1 |  |  |
| *M. digitata* |  | 33 | C15 |  |  |
| *M. digitata* |  | 35 | C1 |  |  |
| *M. digitata* |  | 82 | C33 |  |  |
| *M. digitata* |  | 21 | C1 |  |  |
| *M. digitata* |  | 32 | A2 |  |  |
| *M. digitata* |  | 40 | A | “uncultured”/C1 |  |
| *M. digitata* |  | 68 | A3 |  |  |
| *M. digitata* |  | 119 | A |  |  |
| *M. digitata* |  | 28 | C15 |  |  |
| *M. digitata* |  | 104 | C1 |  |  |
| *M. digitata* |  | 39 | C |  |  |
| *M. digitata* |  | 103 | C15 |  |  |
| *M. digitata* |  | 37 | C | A1 |  |
| *M. digitata* |  | 54 | C1 |  |  |
| *M. digitata* |  | 41 | A | “uncultured”/*S. voratum* |  |
| *M. digitata* |  | 69 | A |  |  |
| *M. digitata* |  | 67 | G3 |  |  |
| *M. digitata* |  | 72 | G |  |  |
| *M. digitata* |  | 47 | C |  |  |
| *M. digitata* |  | 70 | A |  |  |
| *M. digitata* |  | 61 | A |  |  |
| *M. digitata* |  | 56 | A2 |  |  |
| *M. digitata* |  | 75 | A |  |  |
| *M. digitata* |  | 43 | C15 |  |  |
| *M. digitata* |  | 76 | C15 |  |  |
| *M. digitata* |  | 45 | C1 |  |  |
| *M. digitata* |  | 57 | C1 |  |  |
| *M. digitata* |  | 3 | D1 |  |  |
| *M. digitata* |  | 71 | C1 |  |  |
| *M. digitata* |  | 49 | C1 |  |  |
| *M. digitata* |  | 55 | C |  |  |
| *M. digitata* |  | 73 | C1 |  |  |
| *M. digitata* |  | 90 | A2 |  |  |
| *M. digitata* |  | 31 | B1 |  |  |
| *M. digitata* |  | 38 | A1 |  |  |
| *M. digitata* |  | 2 | C1 |  |  |
| *M. digitata* |  | 5 | A3 |  |  |
| *M. digitata* |  | 8 | C1 |  |  |
| *M. digitata* |  | 13 | C1 |  |  |
| *M. digitata* |  | 50 | C15 |  |  |
| *M. digitata* |  | 112 | C15 |  |  |
| *M. digitata* |  | 4 | A |  |  |
| *M. digitata* |  | 74 | A | “uncultured”/C1 |  |


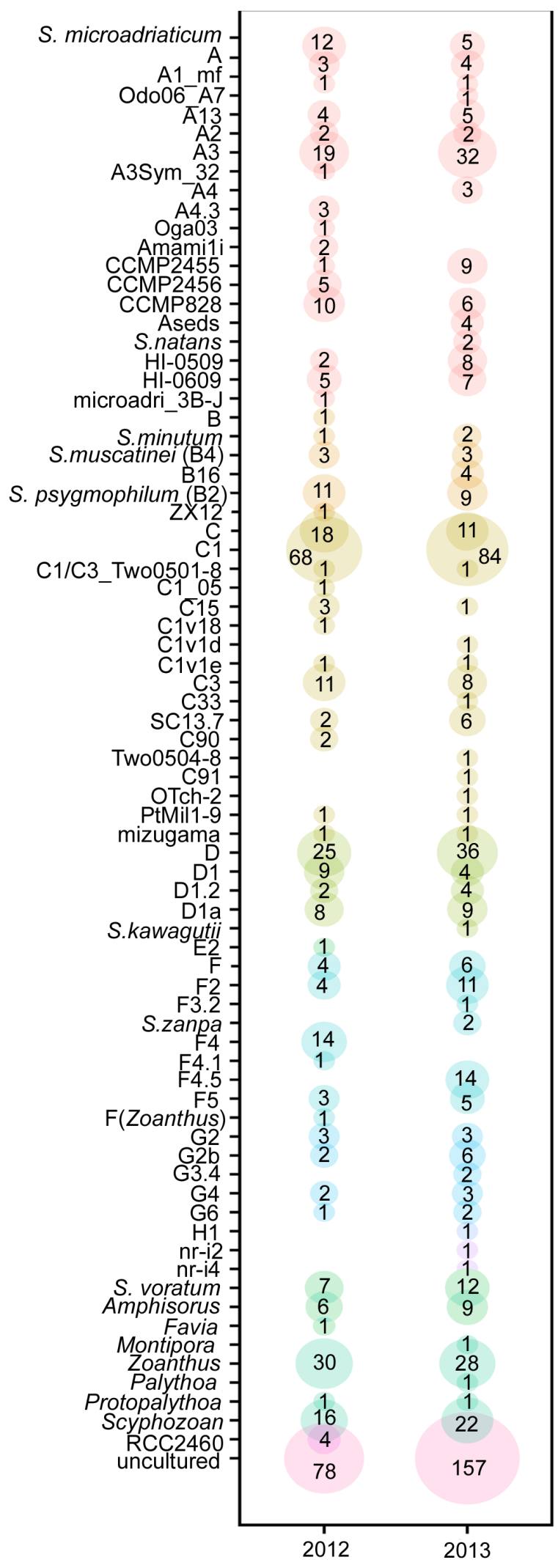


**Figure S1.** Type level *Symbiodinium* diversity retrieved per year. Colours represent the clade designations and the size of the circles represent the number OTUs collapsed per type. Circle diameters are proportional to the number of OTUs. The first column represents OTUs sampled from juveniles in 2012 and the second represents OTUs sampled from juveniles in 2013. For figure clarity, some type have been combined: specifically, category A2 represents OTUs matching A2 and A2_JCUSG-1, Asand (Asand_Oku17, Oku03_sand, Oku16_sand), CCMP2455 (CCMP2455, CCMP2457), *S. psygmophilum* (*psygmophilum*, *psygmophilum*_B2_1635, *psygmophilum*_B2_1636, *psygmophilum*_B2_1637, *psygmophilum*_B2_1638, *psygmophilum*_B2_1639), B16 (B16, B16_Z1), F (12979,1341,13467,13478,1363,1631,1679,1681), and “uncultured” (“uncultured”, zoox21, zoox23, OTU7, OTU18, OTU28, 04-218-SCI.01).

**
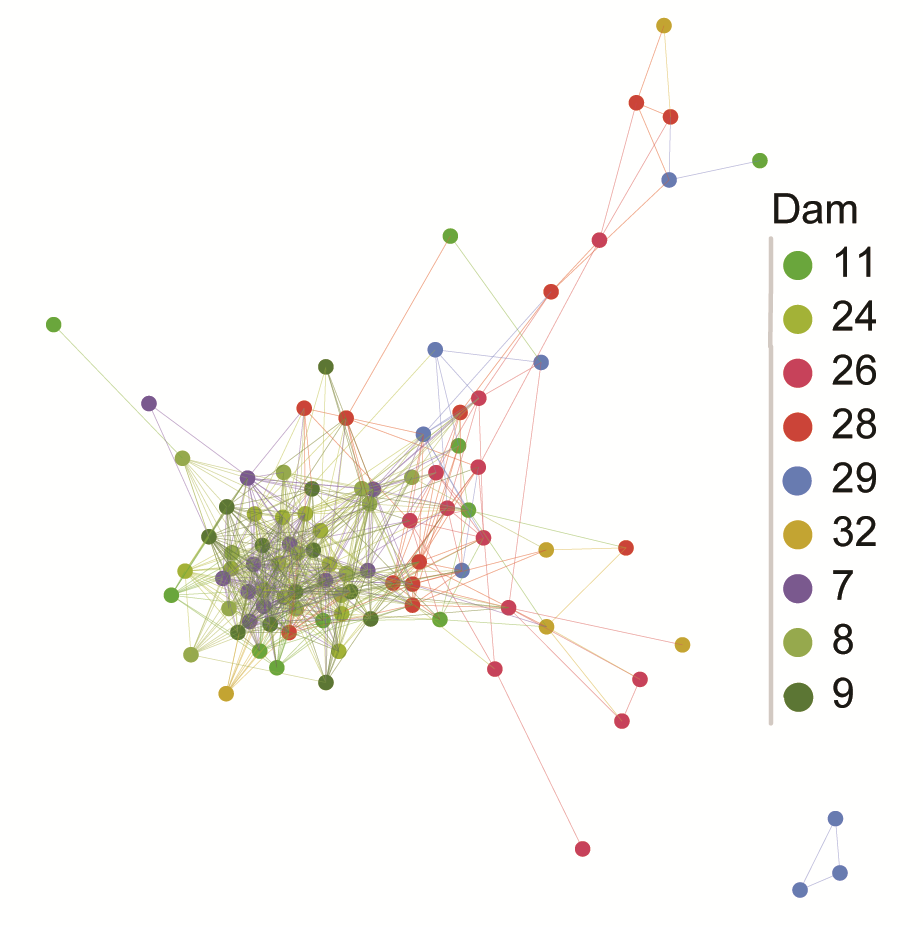
**

**Figure S2.** Network analysis incorporating unweighted Unifrac distances of *Symbiodinium* diversity in *Montipora digitata* eggs. Each point represents a single egg sample. Colours correspond to dam designations in Fig. 3.

**
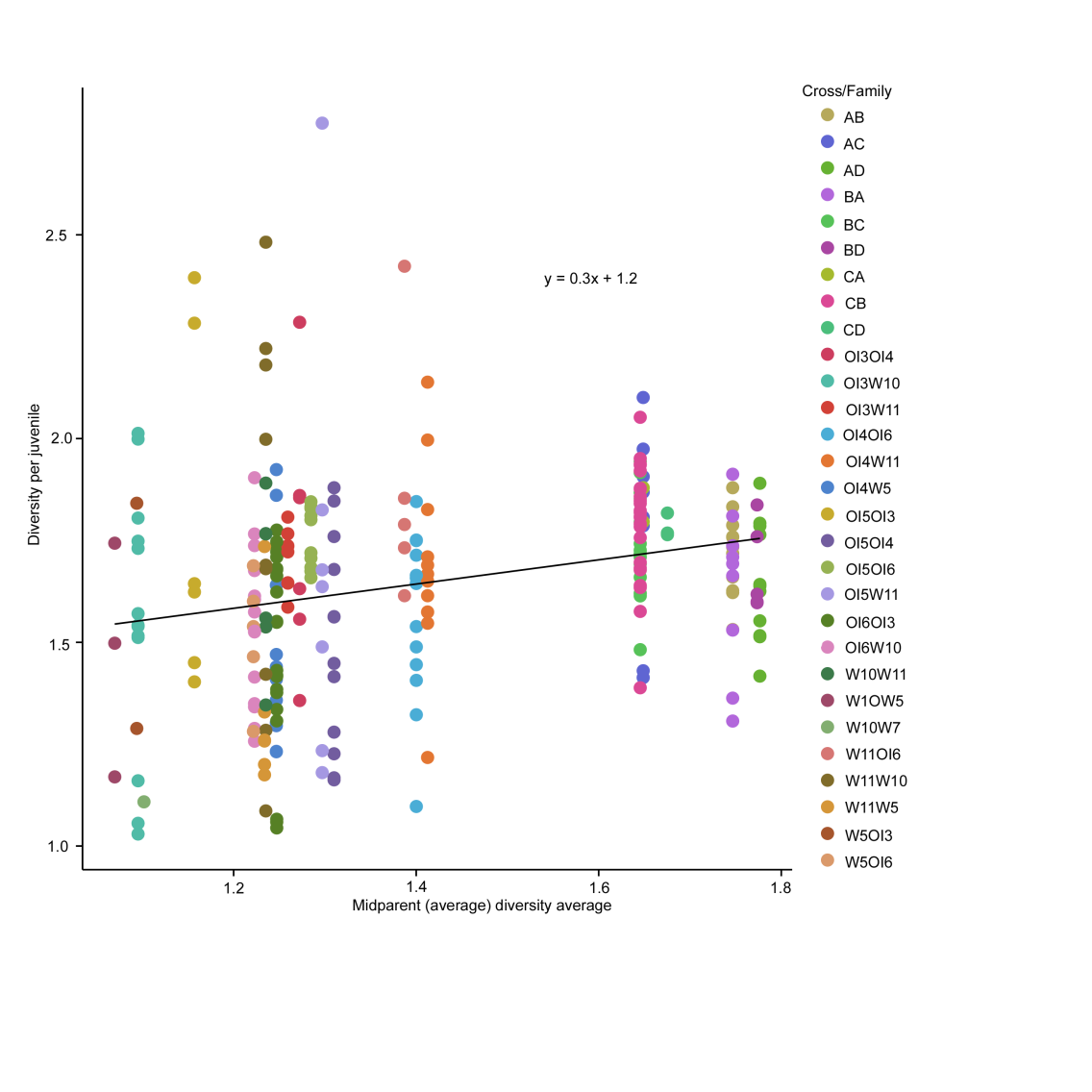
**

**Figure S3.** Heritability estimation, as calculated from the regression of the average *Symbiodinium* diversity metric for each *A. tenuis* juvenile against the average *Symbiodinium* diversity metric for each juvenile’s dam and sire. The slope of the regression line is equivalent to the narrow-sense heritability (h^2^).

**
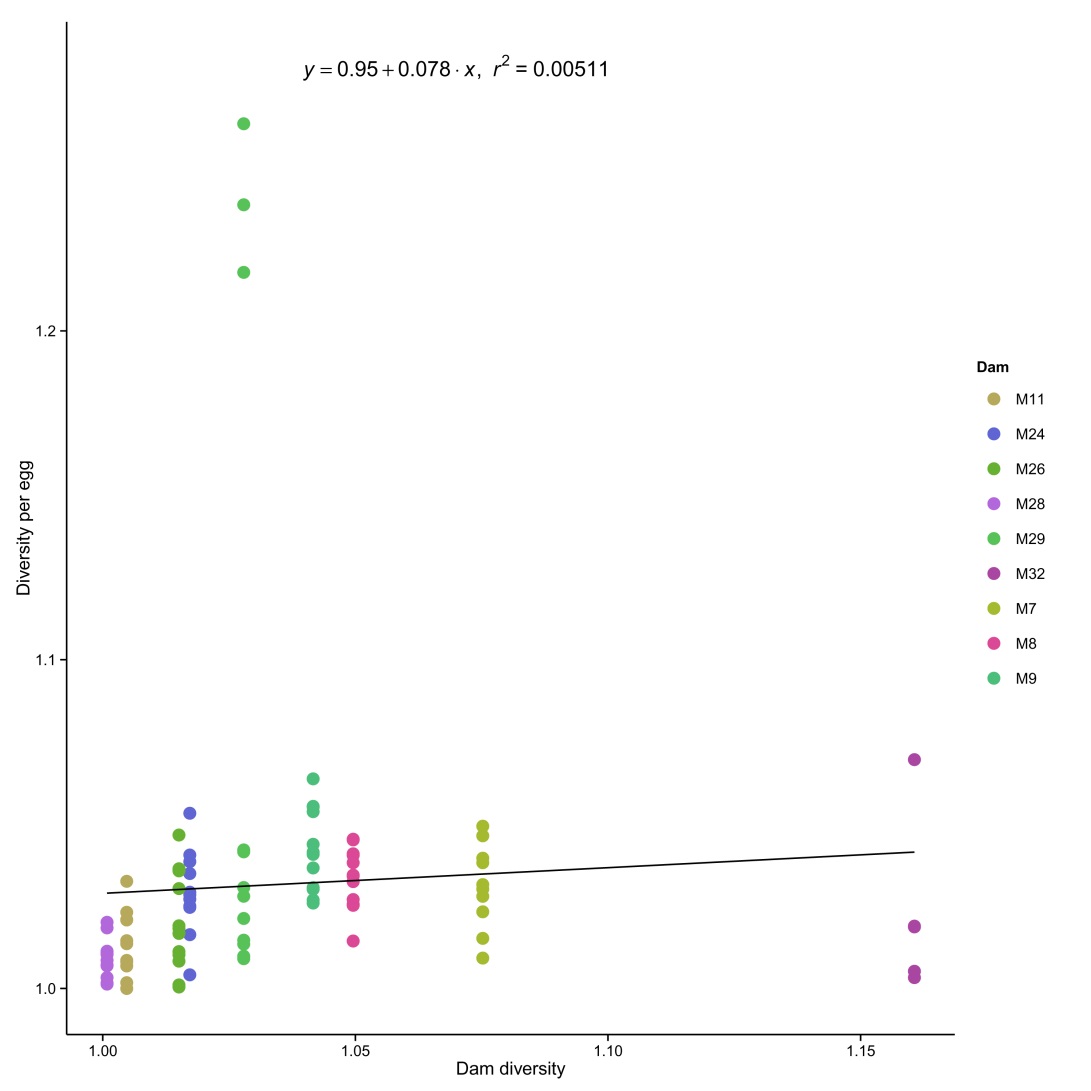
**

**Figure S4.** Heritability estimation, as calculated from the regression of the average *Symbiodinium* diversity metric for each *M. digitata* egg against the average *Symbiodinium* diversity metric for dams. The slope of the regression line is equivalent to the narrow-sense heritability (h^2^) multiplied by 2 as only a single parent is represented on the x-axis.
